# Wave–Particle–Entanglement–Ignorance Complementarity for General Bipartite Systems

**DOI:** 10.3390/e22080813

**Published:** 2020-07-24

**Authors:** Wei Wu, Jin Wang

**Affiliations:** 1 State Key Laboratory of Electroanalytical Chemistry, Changchun Institute of Applied Chemistry, Chinese Academy of Sciences, Changchun 130022, China; weiwuhere@ciac.ac.cn; 2 Department of Chemistry, State University of New York at Stony Brook, Stony Brook, NY 11794, USA; 3 Department of Physics and Astronomy, State University of New York at Stony Brook, Stony Brook, NY 11790, USA

**Keywords:** complementarity, wave–particle duality, entanglement, predictability, visibility, 03.65.Ta, 03.65.Ud, 03.67.–a

## Abstract

Wave–particle duality as the defining characteristic of quantum objects is a typical example of the principle of complementarity. The wave–particle–entanglement (WPE) complementarity, initially developed for two-qubit systems, is an extended form of complementarity that combines wave–particle duality with a previously missing ingredient, quantum entanglement. For two-qubit systems in mixed states, the WPE complementarity was further completed by adding yet another piece that characterizes ignorance, forming the wave–particle–entanglement–ignorance (WPEI) complementarity. A general formulation of the WPEI complementarity can not only shed new light on fundamental problems in quantum mechanics, but can also have a wide range of experimental and practical applications in quantum-mechanical settings. The purpose of this study is to establish the WPEI complementarity for general multi-dimensional bipartite systems in pure or mixed states, and extend its range of applications to incorporate hierarchical and infinite-dimensional bipartite systems. The general formulation is facilitated by well-motivated generalizations of the relevant quantities. When faced with different directions of extensions to take, our guiding principle is that the formulated complementarity should be as simple and powerful as possible. We find that the generalized form of the WPEI complementarity contains unequal-weight averages reflecting the difference in the subsystem dimensions, and that the tangle, instead of the squared concurrence, serves as a more suitable entanglement measure in the general scenario. Two examples, a finite-dimensional bipartite system in mixed states and an infinite-dimensional bipartite system in pure states, are studied in detail to illustrate the general formalism. We also discuss our results in connection with some previous work. The WPEI complementarity for general finite-dimensional bipartite systems may be tested in multi-beam interference experiments, while the second example we studied may facilitate future experimental investigations on complementarity in infinite-dimensional bipartite systems.

## 1. Introduction

**Figure 1:**
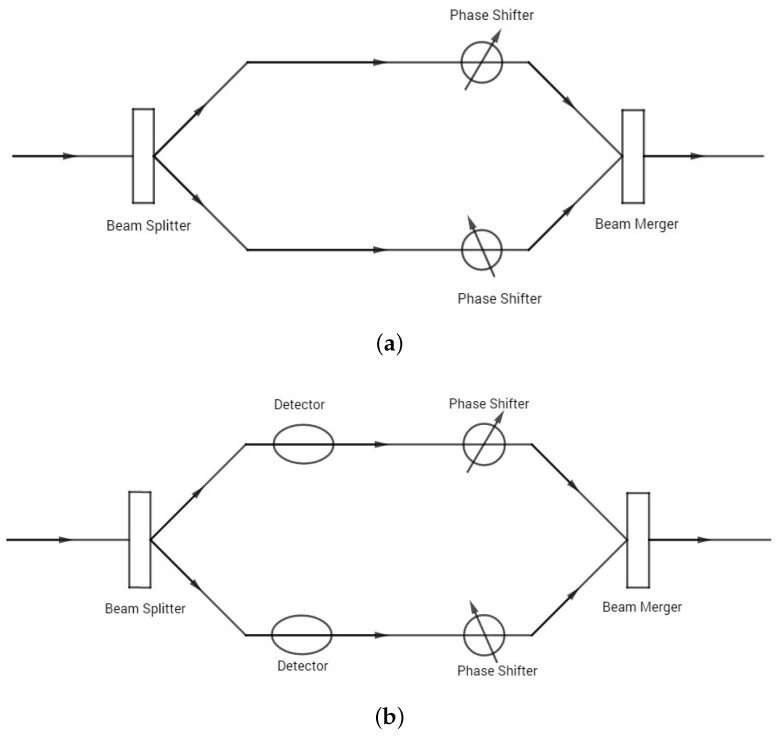
Schematic two-beam interferometer. (**a**) two-beam interferometer without which-way detectors. The input beam is split into two beams by the beam splitter, phase-shifted by the phase shifters, and then recombined by the beam merger to produce the output beam; (**b**) two-beam interferometer with which-way detectors. Detectors are placed on the path of each beam to acquire which-way knowledge, adapted from Reference [5].

Wave–particle duality occupies a central position in the world of quantum weirdness. It is usually considered a prime example of Bohr’s principle of complementarity, which states that quantum systems (“quantons”) may possess properties that are equally real but mutually exclusive [1]. In the context of wave–particle duality, this means that the full wave nature cannot be observed simultaneously with the full particle nature, although both aspects are indispensable for a complete description of the quantum reality. Attempts have been made to develop quantitative formulations of wave–particle duality in two-beam interference experiments [2,3,4,5]. A brief review of the relevant history can be found in Reference [6]. In these efforts, quantitative measures of wave and particle properties were constructed and constrained in mathematical relations in accordance with quantum mechanics. Accompanying the quantitative formulation was the expansion of the content of wave–particle duality, from the extreme cases of full wave and particle natures existing in mutual exclusion, to the intermediate cases of partial wave and particle natures coexisting in compliance with a compatibility relation. Two basic versions of quantitative wave–particle duality relations were proposed [3,4,5]. They correspond to two different setups of two-beam interference experiments, with or without which-way detectors (devices detecting which way the quanton takes). The two setups are schematically displayed in Figure 1.

In the first setup without which-way detectors shown in Figure 1a, the input beam is split into two beams by the beam splitter. Then, the phase of each beam is shifted by the phase shifter on each path, after which the two beams are recombined to produce the output beam for measurements. Wave–particle duality in this setup can be quantified by the inequality [3,4,5]:

(1) $$P^{2}+V^{2}\leq 1,$$

where *P* is the path predictability quantifying the particle property, and *V* is the visibility of interference fringes measuring the wave property. The fringe visibility has the standard expression *V* = $(I_{\max}$ − $I_{\min})/\left(I_{\max}+I_{\min}\right)$, where $I_{\max}$ and $I_{\min}$ are the maximum and minimum intensities of the output beam. It quantifies the degree of contrast of the interference pattern. Given that interference is a signature of waves, it is reasonable to use the fringe visibility to quantify the wave nature. On the other hand, the path predictability needs a little more explanation. The path predictability measures the ability to predict which path the quanton takes, or a priori which-way knowledge. Assume that the two beams (beam 1 and beam 2) in the interferometer have intensities $I_{1}$ and $I_{2}$, respectively. (Note that the phase shifters do not affect $I_{1}$ or $I_{2}$, as they only modulate the phases of the beams.) Then, it is known a priori that a quanton coming out of the beam splitter has the probability $p_{1}=I_{1}/\left(I_{1}+I_{2}\right)$ to be in beam 1 and $p_{2}=I_{2}/\left(I_{1}+I_{2}\right)$ to be in beam 2. The path predictability, $P=|p_{1}-p_{2}\left|=\right|\left(I_{1}-I_{2}\right)/\left(I_{1}+I_{2}\right)|$, thus measures the ability to predict which way the quanton goes through, based on the distinction in the a priori probabilities of the two paths. If one bets on the more probable way, then the probability to win the bet is (1+P)/2 [5]. In the special case of a symmetric interferometer with $I_{1}=I_{2}$, or $p_{1}=p_{2}$, the path predictability vanishes, and there is no way to tell beforehand which way the quanton will take (or is more likely to take). Since having a trajectory (path or way) is typical of particles, the path predictability is a legitimate measure of the particle nature. The wave–particle duality relation, in the form of $P^{2}+V^{2}\leq 1$, then limits the extent to which both the a priori which-way knowledge can be gained (particle nature) and the interference pattern can be observed (wave nature).

**Figure 2:**
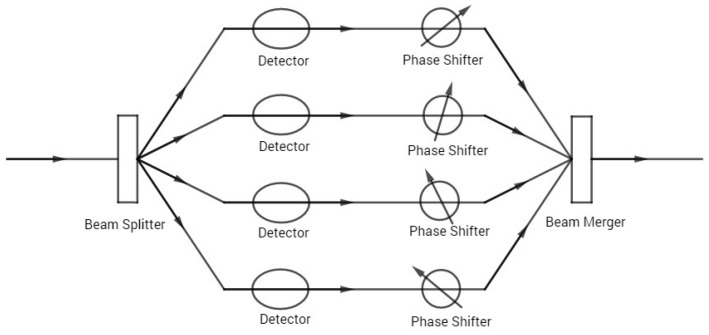
Schematic four-beam interferometer. Detectors are placed on the path of each beam to acquire which-way knowledge.

In the second setup of the interference experiment in Figure 1b, detectors are placed on the path of each beam to acquire which-way knowledge through interaction with the quanton. In this setup, quantitative wave–particle duality relation has the form [5]

(2) $$D^{2}+V^{2}\leq 1,$$

where *D* is the path distinguishability, a measure of the particle nature, and *V* is still the fringe visibility, a measure of the wave nature. Although formally similar, the wave–particle duality relation in Equation (2) has a very different interpretation from that in Equation (1). The distinction lies in how the particle nature is characterized. In contrast with the path predictability *P* that represents the a priori which-way knowledge, the path distinguishability *D* represents the a posteriori which-way knowledge, gained retrodictively from measurements performed with which-way detectors interacting with the quanton. As a result, the path distinguishability is not only determined by the quanton, but also depends on the detector [5]. By contrast, the path predictability is determined only by the state of the quanton (related to the setup of the interferometer). The wave–particle duality relation, in the form of $D^{2}+V^{2}\leq 1$, sets a limit on the extent to which the a posteriori which-way knowledge gained with detectors (particle nature) is compatible with the observation of the interference pattern (wave nature). Hence, the message it conveys is different from that in Equation (1). There has been effort to generalize both versions of the wave–particle duality relations to multi-beam interference experiments (corresponding to quantum systems with multi-dimensional Hilbert spaces) [7,8,9,10,11,12,13,14]. A schematic four-beam interferometer is shown in Figure 2.

Another direction that has enriched the physical content of the complementarity principle is the investigation of wave–particle duality in relation to quantum entanglement [15,16,17,18,19]. In particular, Jakob and Bergou extended the wave–particle duality relation to a wave–particle–entanglement (WPE) “triality” relation for general two-qubit systems (bipartite two-state systems) [15]. When the two-qubit system is in a pure state, the *WPE complementarity relation* has the form of a tight equality involving three quantities:

(3) $$P_{k}^{2}+V_{k}^{2}+C^{2}=1,$$

where k=1 or 2 indicates the individual qubit, $P_{k}$ is the path predictability of qubit *k* measuring its particle aspect, $V_{k}$ is the fringe visibility of qubit *k* characterizing its wave aspect, and *C* is the concurrence quantifying the entanglement between the two qubits. The wave–particle duality relation for each qubit in the form of $P_{k}^{2}+V_{k}^{2}\leq 1$ is obviously implied by this WPE triality relation. The predictability $P_{k}$ and the visibility $V_{k}$ are single-partite (local) properties, while the concurrence *C* is a bipartite (nonlocal) property involving both qubits. The single-partite properties, predictability $P_{k}$ and visibility $V_{k}$, can be combined to form another single-partite quantity $Q_{k}$ defined by

(4) $$P_{k}^{2}+V_{k}^{2}=Q_{k}^{2}.$$

(The quantity $Q_{k}$ was denoted by $S_{k}$ in Reference [15]. To avoid confusion with the linear entropy to be introduced later, we used a different notation.) $Q_{k}$ is invariant under local unitary transformations, while $P_{k}$ and $V_{k}$ are not. The local invariant $Q_{k}$ is a quantitative measure of the *quantum* nature of the single-partite reality, which consists of two complementary aspects, the particle aspect quantified by the predictability $P_{k}$ and the wave aspect captured by the visibility $V_{k}$. If $Q_{k}$ is held constant, then Equation (4) implies the complementarity between $P_{k}$ (particle) and $V_{k}$ (wave), in the sense that the increase of one necessarily implies the decrease of the other. Hence, this is a statement of wave–particle duality in quantum reality. Given that all three quantities in Equation (4) are local (single-partite) properties, Equation (4) may be referred to as the *local complementarity relation*. Then, the WPE complementarity relation in Equation (3) also implies another complementarity relation

(5) $$Q_{k}^{2}+C^{2}=1,$$

which relates the local (single-partite) quantum property characterized by $Q_{k}$ to the nonlocal (bipartite) quantum property quantified by the entanglement measure *C* in a complementary way, reflecting a trade-off between local and nonlocal quantum properties. In this regard, Equation (5) may be referred to as the *local-nonlocal complementarity relation*. The WPE complementarity relation in Equation (3) is equivalent to the combination of the local complementarity relation in Equation (4) and the local-nonlocal complementarity relation in Equation (5). In addition, when the two-qubit system is in a mixed state, the WPE complementarity relation becomes an inequality

(6) $$P_{k}^{2}+V_{k}^{2}+C^{2}\leq 1.$$

By identifying the path distinguishability $D_{k}^{2}=P_{k}^{2}+C^{2}$, the wave–particle duality in the form of $D_{k}^{2}$ + $V_{k}^{2}$ ≤ 1 can also be recovered for the two-qubit system.

A further development of the complementarity for two-qubit systems in mixed states is completed by Tessier [20]. The local-nonlocal complementarity relation in Equation (5) for pure states was generalized to mixed states of two-qubit systems, which, in our notations, reads [20]

(7) $$\bar{Q_{k}^{2}}+C^{2}+B^{2}=1,$$

where $\bar{Q_{k}^{2}}=\left(Q_{1}^{2}+Q_{2}^{2}\right)/2$, and $\eta =B^{2}\in \left[0,1\right]$ is referred to as “separable uncertainty” in Reference [20]. We shall refer to *B* instead of $B^{2}$ as separable uncertainty in this article, which represents the *ignorance* in the states of the two individual qubits, originating from the mixedness of the two-qubit state rather than the entanglement between the two qubits. (The meaning of the separable uncertainty will be further discussed later.) When the two-qubit system is in a pure state, B=0 and $Q_{1}=Q_{2}$ so that Equation (7) reduces to Equation (5) for pure states. In terms of Equation (7), the WPE complementarity is extended to a general mixed state of two-qubit systems, which yet again takes the form of a tight equality

(8) $$\bar{P_{k}^{2}}+\bar{V_{k}^{2}}+C^{2}+B^{2}=1,$$

where $\bar{P_{k}^{2}}=\left(P_{1}^{2}+P_{2}^{2}\right)/2$ and $\bar{V_{k}^{2}}=\left(V_{1}^{2}+V_{2}^{2}\right)/2$ are equal-weight averages of the single-partite properties. Given its structure, we shall refer to this complementarity relation in Equation (8) as the *wave–particle–entanglement–ignorance (WPEI) complementarity*. The WPE complementarity in Equation (3) is recovered from Equation (8) when the two-qubit system is in a pure state. The WPE complementarity for pure states in Equation (3) and its generalization to the WPEI complementarity for mixed states in Equation (8) represent a more complete formulation of the complementarity principle for two-qubit systems.

The complementarity relations in Equations (3)–(5) have also been recently derived in the context of classical polarized optical field [21,22,23], where the degree of polarization plays the role of the single-partite quantum property *Q*, the polarization coherence theorem [22] is the counterpart of the local complementarity relation in Equation (4), and the coherent constraint [23] corresponds to the local-nonlocal complementarity relation in Equation (5). (What was termed distinguishability in References [21,22,23] is, strictly speaking, predictability.) The WPE complementarity has been further investigated in relation to multi-partite entanglement in multi-qubit systems [20,24,25]. There is a simplification in multi-qubit systems as any single qubit is considered in relation to the rest. The single-partite properties (*P*, *V* and *Q*) for a single qubit apply directly without any change. The bipartite entanglement between the qubit and the rest can be quantified by generalized concurrence [26,27,28]. The multi-partite entanglement measure can then be constructed from these bipartite entanglements between a single qubit and the rest. This allows convenient investigation of complementarity relations in multi-qubit systems in connection with multi-partite entanglement, in terms of single-qubit properties and bipartite entanglements. However, for a general bipartite system, each subsystem may have multiple states with a multi-dimensional Hilbert space. As a result, the simplification in multi-qubit systems no longer applies and the problem needs to be addressed in a more direct way. A multi-beam interference experiment with which-way detectors, as schematically shown in Figure 2, may be considered as a typical example of such multi-dimensional bipartite systems. One subsystem is the quanton in the interferometer, whose state in the interferometer is described by a multi-dimensional density matrix. The other subsystem is the which-way detector with multiple states in general (the detectors on the individual paths may be interpreted as components of the measurement device collectively termed the which-way detector). The interaction between the quanton and the which-way detector typically brings the bipartite system into an entangled state, even if it was initially prepared in a separable state. In addition, the quanton-detector bipartite system may also interact with an external environment, which brings the quanton-detector system into a mixed state. Therefore, a general formulation of the WPEI complementarity for general multi-dimensional bipartite systems in pure or mixed states is not only of theoretical interest that may shed new light on fundamental problems in quantum mechanics, but also of experimental relevance as it may be tested in these quanton-detector bipartite systems in multi-beam interference experiments. We mention that there has also been effort to extend complementarity beyond the bipartite system settings [29].

The objective of the present work is to formulate the WPEI complementarity (including the WPE complementarity) for general multi-dimensional bipartite systems in pure or mixed states, and push its range of applications to include hierarchical bipartite systems (bipartite systems consisting of bipartite sytems) and infinite-dimensional bipartite systems. The general formulation is facilitated by well-motivated extensions of the relevant properties. In case there are different directions of generalization to take, our guiding principle is that the formulated complementarity relation should be as simple and powerful as possible. We find that the extended form of the WPEI complementarity contains unequal-weight averages arising from the difference in the subsystem dimensions, and that the entanglement measure of the squared concurrence is more suitably replaced by the tangle in the general scenario. We illustrate the general formalism by studying two particular examples worked out in detail. One example is a qubit–qutrit system in rank-2 mixed states and the other is a pair of infinite-dimensional quantons in entangled coherent states. The relations of the present work to some previous study are also discussed. The WPEI complementarity in this work for general bipartite systems in pure or mixed states rests on the basic principles and structures of quantum mechanics, which has a general feature that extends even to hierarchical and infinite-dimensional bipartite systems. It has a wide range of potential applications in various quantum-mechanical settings, including the multi-beam interference experiments and future experimental investigations on hierarchical and infinite-dimensional bipartite systems.

The rest of this article is organized as follows. In Section 2, we formulate the WPEI complementarity for general multi-dimensional bipartite systems, and extend its range of applications to incorporate hierarchical and infinite-dimensional bipartite systems. In Section 3, we study two particular examples in detail, a qubit–qutrit system in rank-2 mixed states, and a pair of infinite-dimensional quantons in entangled coherent states, in order to illustrate the general formulation. The results obtained in the present work are discussed in relation to the previous work in Section 4, with a focus on the issue of measure matching. Finally, the conclusions are given in Section 5.

## 2. WPEI Complementarity for General Bipartite Systems

In this section, we establish the WPEI complementarity relations for general multi-dimensional bipartite systems in pure or mixed states. We first clarify a few points on the two-qubit systems in preparation for the general formulation. The WPE complementarity relations are then developed for general bipartite systems in pure states. We further formulate the WPEI complementarity relations for general bipartite systems in mixed states, and present a graphical representation that encapsulates all the relevant relations in a picture. After establishing the complementarity relations for general bipartite systems, we expand the range of applications of these results by discussing how they can be applied to hierarchical bipartite systems and infinite-dimensional bipartite systems.

### 2.1. Some Clarifications of the Two-Qubit Systems

We clarify a few points on the two-qubit systems that will facilitate the general formulation. The first point is on the physical meaning of the single-partite quantity *Q*. In the qubit system, *Q* is geometrically represented by the length of the Bloch vector of the qubit, which is essentially a (normalized) measure of the *purity* of the single qubit state. Its connection to the usual purity $\mathrm{tr}(\rho^{2})$ is given by the formula:

(9) $$Q\left(\rho \right)=\left|a\right|=\sqrt{2\mathrm{tr}(\rho^{2})-1},$$

where ρ=(I+a·σ)/2 is the density operator of the qubit and a is the Bloch vector. This formula shows that *Q* is a monotonically increasing function of the usual purity $\mathrm{tr}(\rho^{2})$, and it takes values in the range [0,1] while $\mathrm{tr}\left(\rho^{2}\right)\in \left[1/2,1\right]$. Therefore, *Q* measures the degree of pureness of the quantum state of the qubit. Pure states have maximum purity Q=1, while mixed states have less purity. Mixed states may arise from a statistical mixture of pure quantum states (i.e., ignorance) or entanglement with another quantum system. In either case, mixed states contain a certain level of missing information on the state of the quantum system under consideration. The missing information degrades the purity of the quantum state of the system, resulting in less purity for mixed states. Pure states are the most informative description of the state of the system compatible with quantum mechanics. Thus, in a certain sense, the purity *Q* also quantifies the amount of “quantum information”, or the degree of “quantumness”, in the state of the system. In addition, the local complementarity relation, $P^{2}+V^{2}=Q^{2}$, also shows that *Q* determines the level of wave–particle duality, a quantum signature. In particular, if Q=0 (for the maximally mixed state ρ=I/2), then P=0 and V=0. That means neither particle aspect (predictability) nor wave aspect (visibility) can be observed. Thus, there is nothing quantum in such a state with Q=0. All these justify that *Q* is a quantitative measure of the *quantum* nature of the single-partite reality. The notation *Q* is also used as a reminder of this quantum aspect in this meaning. *Q* may be termed quantum purity to stress both the quantum nature and the purity measure. For simplicity, we also just call it purity in this work, with the quantum aspect encoded in the notation *Q*. In case possible confusion may arise, $\mathrm{tr}(\rho^{2})$ is referred to as the “usual purity”.

Furthermore, with *Q* identified as the (quantum) purity of the single qubit, the local-nonlocal complementarity relation for pure states of the two-qubit system, $Q_{k}^{2}+C^{2}=1$, has the meaning that the concurrence measuring the entanglement between the two qubits is complementary to the purity of the single qubit. In other words, the more entangled the two qubits are, the less pure (i.e., more mixed) each single qubit is. In fact, the concurrence for a two-qubit pure state |Ψ〉 has the expression:

(10) $$C\left(\Psi \right)=\sqrt{S_{L}\left(\rho_{k}\right)}=\sqrt{2[1-\mathrm{tr}\left(\rho_{k}^{2}\right)]},$$

where $\rho_{k}$ (k=1,2) is the reduced density operator of either single qubit, and $S_{L}\left(\rho_{k}\right)=2\left[1-\mathrm{tr}\left(\rho_{k}^{2}\right)\right]$ is the (normalized) linear entropy that measures the degree of impurity (i.e., mixedness) of the state $\rho_{k}$. In the current context, the square root of the linear entropy may be a more natural quantity to use, which we define as the mixedness:

(11) $$M\left(\rho_{k}\right)=\sqrt{S_{L}\left(\rho_{k}\right)}=\sqrt{2[1-\mathrm{tr}\left(\rho_{k}^{2}\right)]}.$$

The mixedness *M* is also a *single-partite* property quantifying the degree of mixedness (i.e., impurity) of the quantum state as the linear entropy $S_{L}$ is. In addition, as suggested by its connection to the linear *entropy*, *M* also measures the level of missing quantum information in the state. The mixedness *M* is the exact opposite of the purity *Q*, and they satisfy the *purity–mixedness complementarity relation*:

(12) $$Q_{k}^{2}+M_{k}^{2}=1,$$

where $Q_{k}\equiv Q\left(\rho_{k}\right)$ and $M_{k}\equiv M\left(\rho_{k}\right)$. This is a completely local, single-partite relation, in contrast with the local-nonlocal complementarity relation $Q_{k}^{2}+C^{2}=1$ for pure states. In terms of the mixedness, Equation (10) simply becomes

(13) $$C\left(\Psi \right)=M_{k}.$$

That is, the concurrence measuring the entanglement between the two qubits in a pure state is equal to the mixedness of either single qubit. Although numerically the same, it is important to notice that conceptually the concurrence as an entanglement measure is a bipartite property, while the mixedness is a single-partite property that can be defined without referring to another system.

In addition, when the two-qubit system is in a mixed state, the concurrence *C* of the two-qubit system in general is no longer equal to the mixedness of the single qubit. In fact, the combination of the purity–mixedness complementarity in Equation (12) with the local-nonlocal complementarity for mixed states in Equation (7) yields the following *entanglement–mixedness relation* for mixed states [20]:

(14) $$C^{2}+B^{2}=\bar{M_{k}^{2}},$$

where $\bar{M_{k}^{2}}=\left(M_{1}^{2}+M_{2}^{2}\right)/2$. The r.h.s. of this equation, $\bar{M_{k}^{2}}$, consisting of the mixedness of the two individual qubits, characterizes the level of missing quantum information in the states of the two individual qubits. The l.h.s. of the equation shows that the missing quantum information originates from two sources. One source is the entanglement between the two qubits and the other source can be identified as the ignorance associated with the mixedness of the two-qubit state. (The latter could still originate from entanglement with another system or ignorance.) The separable uncertainty *B* is a measure of the ignorance in the states of the individual qubits associated with the mixedness of the two-qubit state rather than the entanglement between the two qubits. It is invariant under the local unitary transformations, as seen from the formula $B^{2}=\bar{M_{k}^{2}}-C^{2}$. Later, we shall show the explicit connection between the separable uncertainty and the mixedness of the bipartite state for rank-2 states. In the special case when the two-qubit system is in a pure state, we have B=0 and $M_{1}=M_{2}$. As a result, the relation $C\left(\Psi \right)=M_{k}$ in Equation (13) for pure states is recovered from the more general entanglement–mixedness relation in Equation (14).

### 2.2. Generalized WPE Complementarity for Pure States of General Bipartite Systems

We first formulate the WPE complementarity relations for general multi-dimensional bipartite systems in pure states. This requires appropriate extensions of the involved properties to multi-dimensional systems.

#### 2.2.1. Generalized Local-Nonlocal Complementarity for Pure States

Rungta et al. [26,27] generalized the concurrence to arbitrary finite-dimensional systems. The generalized concurrence for bipartite systems in a pure state |Ψ〉 is given by [26]

(15) $$C\left(\Psi \right)=\sqrt{2[1-\mathrm{tr}\left(\rho_{k}^{2}\right)]},$$

where $\rho_{k}$ (k=1,2) is the reduced density operator of either subsystem in the bipartite system. C(Ψ) is not normalized (except for two-qubit systems); it takes values in the range $\left[0,\sqrt{2\left(n_{\min}-1\right)/n_{\min}}\right]$, where $n_{\min}=\min\left\{n_{1},n_{2}\right\}$ is the lower dimension of the two subsystems [27]. It may be tempting to normalize C(Ψ) so that it is in the range [0,1]. However, the normalization factor depends on $n_{\min}$, which is not symmetric with respect to the two subsystems if they have different dimensions, i.e., $n_{1}\neq n_{2}$. Hence, it is not very useful to normalize C(Ψ) when it is considered in relation to both subsystems with $n_{1}\neq n_{2}$. We shall use C(Ψ) in Equation (15) without normalization.

Similar to the two-qubit system case, the generalized concurrence for bipartite systems in pure states is closely related to the linear entropy of the subsystems. We consider this issue in a more general context. For a general *n*-dimensional quantum system with the density operator ρ, the (normalized) linear entropy is defined as

(16) $$S_{L}\left(\rho \right)=\frac{n}{n-1}\left[1-\mathrm{tr}\left(\rho^{2}\right)\right],$$

where $\mathrm{tr}(\rho^{2})$ is the usual purity. We also define the square root of the linear entropy as the (normalized) mixedness, namely,

(17) $$M\left(\rho \right)=\sqrt{S_{L}\left(\rho \right)}=\sqrt{\frac{n}{n-1}\left[1-\mathrm{tr}\left(\rho^{2}\right)\right]}.$$

The mixedness M is a single-partite property that measures the degree of mixedness (impurity) of the state ρ just as the linear entropy does. The opposite of the mixedness M is the purity Q, measuring the degree of purity of the state ρ. The (normalized) purity can be defined as

(18) $$Q\left(\rho \right)=\sqrt{\frac{n}{n-1}\mathrm{tr}\left(\rho^{2}\right)-\frac{1}{n-1}},$$

which is a monotonically increasing function (ranging from 0 to 1) of the usual purity $\mathrm{tr}\left(\rho^{2}\right)\in \left[1/n,1\right]$. Q reduces to the purity *Q* for the qubit in Equation (9) with n=2. The purity and the mixedness satisfies the purity–mixedness complementarity relation

(19) $$Q^{2}+M^{2}=1.$$

Equations (16)–(19) apply to a general *n*-dimensional quantum system. When specified to each subsystem of the bipartite system, Equation (19) reads $Q_{k}^{2}+M_{k}^{2}=1$, where $Q_{k}\equiv Q\left(\rho_{k}\right)$, $M_{k}$ ≡ $M(\rho_{k})$ and *n* is replaced with $n_{k}$ in the expressions.

For general bipartite systems in pure states, according to Equations (15) and (17), the generalized concurrence is related to the mixedness of the subsystems as follows:

(20) $$C\left(\Psi \right)=\nu_{k}M_{k},$$

where $\nu_{k}=\sqrt{2\left(n_{k}-1\right)/n_{k}}$. Combined with the purity–mixedness complementarity $Q_{k}^{2}+M_{k}^{2}=1$, we obtain the local-nonlocal complementarity relation for general bipartite systems in pure states

(21) $$Q_{k}^{2}+C^{2}/\nu_{k}^{2}=1.$$

This is the extension of the local-nonlocal complementarity relation for two-qubit systems in pure states in Equation (5), which reduces to the latter in the special case $n_{k}=2$ ($\nu_{k}=1$). It states that, when the bipartite system is in a pure state, there is a trade-off between the nonlocal bipartite quantum property of the composite system (the quantum entanglement quantified by C) and the local single-partite quantum property of the subsystem (the quantum purity measured by $Q_{k}$). The nonlocal quantum property (entanglement) can only be increased at the expense of the local quantum property (purity) and vice versa.

#### 2.2.2. Generalized Local Complementarity

Then, we generalize the local complementarity relation, $P_{k}^{2}+V_{k}^{2}=Q_{k}^{2}$, to multi-dimensional systems. Still, we study this problem in a more general context by considering a general *n*-dimensional system, which can later be specified to be a subsystem of the bipartite system. The generalized purity Q has been given in Equation (18). We still need to extend the predicability and the visibility to a general *n*-dimensional system. Notice that, for qubits, the predictability $P=|\rho_{11}-\rho_{22}|$ is only determined by the diagonal elements of the density matrix, while the visibility $V=2|\rho_{12}|$ is only determined by the off-diagonal elements of the density matrix. The generalized definitions of the predictability and the visibility for multi-dimensional systems are expected to retain these generic features [7,8].

According to the expression of the purity Q in Equation (18), we have

(22) $$Q^{2}=\frac{n}{n-1}\mathrm{tr}\left(\rho^{2}\right)-\frac{1}{n-1}=\frac{n}{n-1}\sum_{ij}|\rho_{ij}{|}^{2}-\frac{1}{n-1}=\frac{n}{n-1}\left(\sum_{i}\rho_{ii}^{2}-\frac{1}{n}\right)+\frac{n}{n-1}\sum_{i\neq j}{\left|\rho_{ij}\right|}^{2},$$

where we simply separated the diagonal and off-diagonal elements of the density matrix. The two terms that compose $Q^{2}$ in Equation (22) suggest that the generalized predictability quantifying the particle nature can be defined as

(23) $$P=\sqrt{\frac{n}{n-1}\left(\sum_{i}\rho_{ii}^{2}-\frac{1}{n}\right)}$$

and the generalized visibility measuring the wave nature can be defined as

(24) $$V=\sqrt{\frac{n}{n-1}\sum_{i\neq j}{\left|\rho_{ij}\right|}^{2}}.$$

It is easy to check that these definitions reduce to those for the qubit system with n=2. These definitions of the predictability and the visibility were previously introduced by Dürr [7]. What we have done here is to motivate their definitions in relation to the generalized purity Q, in the context of the local complementarity relation for multi-dimensional systems

(25) $$P^{2}+V^{2}=Q^{2}.$$

This relation shows that the particle aspect measured by the (generalized) predictability and the wave aspect quantified by the (generalized) visibility are two complementary aspects of a single *quantum* property, the (generalized) purity Q. The purity Q quantifies the amount of “quantum information”, or the degree of “quantumness”, in the state of the system, and it determines the level of the wave–particle duality. The particle aspect P and the wave aspect V obey a trade-off relation as Q is held constant. When specified to either subsystem of a bipartite system, the local complementarity relation reads $P_{k}^{2}+V_{k}^{2}=Q_{k}^{2}$.

#### 2.2.3. Generalized WPE Complementarity for Pure States

For general bipartite systems in pure states, we have obtained the local-nonlocal complementarity relation

(26) $$Q_{k}^{2}+C^{2}/\nu_{k}^{2}=1$$

and the local complementarity relation

(27) $$P_{k}^{2}+V_{k}^{2}=Q_{k}^{2}.$$

Eliminating the purity $Q_{k}$ from these two relations naturally leads to the generalized WPE complementarity relation for multi-dimensional bipartite systems in pure states:

(28) $$P_{k}^{2}+V_{k}^{2}+C^{2}/\nu_{k}^{2}=1,$$

where $\nu_{k}=\sqrt{2\left(n_{k}-1\right)/n_{k}}$. The particle property characterized by the predictability, the wave property captured by the visibility, and the entanglement between the two subsystems quantified by the concurrence, nicely piece together the complementarity relation in the form of a tight equality. For completeness, we list the expressions of the relevant quantities below:

(29) $$\begin{array}{ccc} P_{k} & = & \sqrt{\frac{n_{k}}{n_{k}-1}\left[\sum_{i}{\left(\rho_{ii}^{k}\right)}^{2}-\frac{1}{n_{k}}\right]}, \end{array}$$

(30) $$\begin{array}{ccc} V_{k} & = & \sqrt{\frac{n_{k}}{n_{k}-1}\sum_{i\neq j}{\left|\rho_{ij}^{k}\right|}^{2}}, \end{array}$$

(31) $$\begin{array}{ccc} Q_{k} & = & \sqrt{\frac{n_{k}}{n_{k}-1}\mathrm{tr}\left(\rho_{k}^{2}\right)-\frac{1}{n_{k}-1}}, \end{array}$$

(32) $$\begin{array}{ccc} M_{k} & = & \sqrt{\frac{n_{k}}{n_{k}-1}\left[1-\mathrm{tr}\left(\rho_{k}^{2}\right)\right]}, \end{array}$$

(33) $$\begin{array}{ccc} C(\Psi ) & = & \sqrt{2[1-\mathrm{tr}\left(\rho_{k}^{2}\right)]}. \end{array}$$

Compared to the WPE complementarity for two-qubit systems in pure states, $P_{k}^{2}+V_{k}^{2}+C^{2}=1$, the WPE complementarity for general bipartite systems in pure states, $P_{k}^{2}+V_{k}^{2}+C^{2}/\nu_{k}^{2}=1$, differs in form only by the factor $1/\nu_{k}^{2}$ in the term $C^{2}$. If the two subsystems have the same dimension so that $\nu_{1}=\nu_{2}$, then this factor is merely due to the fact that the concurrence C is not normalized. However, if the two subsystems have different dimensions so that $\nu_{1}\neq \nu_{2}$, then $P_{k}^{2}+V_{k}^{2}+C^{2}/\nu_{k}^{2}=1$ cannot be brought into the form $P_{k}^{2}+V_{k}^{2}+{C^{\prime }}^{2}=1$ where $C^{\prime }$ does not depend on the subsystem label *k*. (Notice that C is independent of the subsystem label *k*.) Hence, this difference in form of the generalized WPE complementarity is not merely a matter of normalization, but reflects the asymmetry in the two subsystems with different dimensions.

### 2.3. Generalized WPEI Complementarity for Mixed States of General Bipartite Systems

We consider the more general scenario when the multi-dimensional bipartite system is in a mixed state, and extend the WPEI complementarity to this setting.

#### 2.3.1. Entanglement–Mixedness Relation

When the bipartite system is in a mixed state, the generalized concurrence measuring the entanglement between the two subsystems is no longer directly related to the mixedness of the subsystems, since the mixedness of the bipartite system also contributes to the mixedness of the subsystems. For two-qubit systems, the relation between the concurrence and the mixedness is given by Equation (14), $C^{2}+B^{2}=\bar{M_{k}^{2}}$. We extend this entanglement–mixedness relation to general bipartite systems in mixed states.

There is an issue of which entanglement measure to use when it comes to mixed states of general bipartite systems. Although the generalized concurrence seems to be a natural option as a direct generalization from two-qubit systems, there is a subtlety as what appears in the entanglement–mixedness relation, $C^{2}+B^{2}=\bar{M_{k}^{2}}$, is the square of the concurrence (also referred to as the tangle for two-qubit systems [30]). For two qubit systems, this is not an issue, since the tangle is simply the squared concurrence. For general bipartite systems, however, it is not so simple. For general bipartite systems in a mixed state, the concurrence and the tangle can be defined through the convex-roof extension of the pure state measures [27,28]. More specifically, for a general mixed state of the bipartite system described by the density operator ρ, the concurrence is defined as [27,31]

(34) $$C\left(\rho \right)=\underset{\left\{p_{i},|\Psi_{i}\rangle \right\}}{\inf}\left\{\sum_{i}p_{i}C\left(\Psi_{i}\right)\right\},$$

and the tangle is defined as

(35) $$\tau \left(\rho \right)=\underset{\left\{p_{i},|\Psi_{i}\rangle \right\}}{\inf}\left\{\sum_{i}p_{i}C^{2}\left(\Psi_{i}\right)\right\},$$

where the infimum is taken over all ensemble decompositions of the density operator $\rho =\sum_{i}p_{i}|\Psi_{i}\rangle \langle \Psi_{i}|$. In the above, $C(\Psi_{i})$ is the concurrence of the bipartite pure state $|\Psi_{i}\rangle$, with the expression

(36) $$C\left(\Psi_{i}\right)=\sqrt{2\left[1-\mathrm{tr}\left(\rho_{k}^{(i)2}\right)\right]},$$

where $\rho_{k}^{\left(i\right)}$ (k=1,2) are the reduced density operators associated with $|\Psi_{i}\rangle$. (Note that in the literature some authors refer to the squared concurrence $C^{2}\left(\rho \right)$ as the tangle. The definition of the tangle in Equation (35) agrees with those in References [27,31].) For two-qubit systems, the relation $\tau \left(\rho \right)=C^{2}\left(\rho \right)$ is recovered for a general mixed state. However, for general multi-dimensional bipartite systems, the concurrence and the tangle are related by the inequality $C^{2}\left(\rho \right)\leq \tau \left(\rho \right)$, which can be proven using convexity properties [27,31]. In particular, the equality holds for pure states, that is, $C^{2}\left(\Psi \right)=\tau \left(\Psi \right)$, which, however, does not apply to a general mixed state.

The fact that $C^{2}\left(\rho \right)$ and τ(ρ) are not always the same poses the question of which entanglement measure to choose. One may argue that, since the separable uncertainty in $C^{2}+B^{2}=\bar{M_{k}^{2}}$ is not yet defined for general bipartite systems, either entanglement measure may be used by exploiting this ”freedom”. We beg to differ. It is important to use appropriate (matched) measures when properties are considered in relation to each other. Mismatched measures could reduce the power of the formulated relation, a point we will revisit in Section 4 when discussing the issue of measure matching. The study in isotropic states and rank-2 states [27,31] suggests that the tangle τ(ρ), instead of the squared concurrence $C^{2}\left(\rho \right)$, may be the more suitable and powerful measure to employ in the formulation of the entanglement–mixedness relation. In particular, using the tangle as the entanglement measure, we obtain a simple relationship between the separable uncertainty and the mixedness of rank-2 states of the bipartite system, as will be shown later. We take this as evidence to support using the tangle in the entanglement–mixedness relation.

We further motivate the extension of the entanglement–mixedness relation by considering the generalized spin-flipping operation in the study of the generalized concurrence [20,26]. The spin-flipping operation for two-qubit systems [32] was extended to the so-called universal state inversion for a general bipartite system. The universal state inversion (generalized spin-flipping) of the density operator ρ for a bipartite system is given by [26]

(37) $$\tilde{\rho }=I-\rho_{1}⊗I_{2}-I_{1}⊗\rho_{2}+\rho ,$$

where $\rho_{k}$ (k=1,2) are the reduced density operators of the two subsystems. Equation (37) leads to

(38) $$\mathrm{tr}\left(\rho \tilde{\rho }\right)=1-\mathrm{tr}\left(\rho_{1}^{2}\right)-\mathrm{tr}\left(\rho_{2}^{2}\right)+\mathrm{tr}\left(\rho^{2}\right),$$

or, equivalently,

(39) $$\mathrm{tr}\left(\rho \tilde{\rho }\right)+\nu^{2}M^{2}/2=\bar{\nu_{k}^{2}M_{k}^{2}},$$

where $\nu_{k}=\sqrt{2\left(n_{k}-1\right)/n_{k}}$ (k=1,2) as before, $\nu =\sqrt{2(n-1)/n}$ with $n=n_{1}n_{2}$, and the bar notation still represents the equal-weight average, i.e., $\bar{\nu_{k}^{2}M_{k}^{2}}=\left(\sum_{k=1}^{2}\nu_{k}^{2}M_{k}^{2}\right)/2$. Here, M=M(ρ) is the mixedness of the bipartite system, while $M_{k}=M\left(\rho_{k}\right)$ is the mixedness of the subsystem *k*, where the mixedness is defined by Equation (17).

Equation (39) is a natural starting point for the generalization of the entanglement–mixedness relation. Consider the special case when the bipartite system is in a pure state ρ=|Ψ〉〈Ψ|. In this case, we have M=0 and $\nu_{1}M_{1}=\nu_{2}M_{2}$ (due to $\mathrm{tr}\left(\rho_{1}\right)=\mathrm{tr}\left(\rho_{2}\right)$). As a result, Equation (39) reduces to $\mathrm{tr}\left(\rho \tilde{\rho }\right)=\nu_{k}^{2}M_{k}^{2}$ for pure states. Compared with the relation $C\left(\Psi \right)=\nu_{k}M_{k}$ in Equation (20), we see that $\mathrm{tr}\left(\rho \tilde{\rho }\right)=C^{2}\left(\rho \right)=\tau \left(\rho \right)$ for pure states ρ=|Ψ〉〈Ψ|. Hence, Equation (39) recovers the entanglement–mixedness relation for pure states $C\left(\Psi \right)=\nu_{k}M_{k}$, in the equivalent form τ(Ψ) = $C^{2}\left(\Psi \right)$ = $\nu_{k}^{2}M_{k}^{2}$. In this special case, we also see that $\mathrm{tr}(\rho \tilde{\rho })$ for pure states is simply the tangle measuring the entanglement between the two subsystems. When the bipartite system is in a mixed state, $\mathrm{tr}(\rho \tilde{\rho })$ is no longer the tangle that quantifies the entanglement, but it is still closely related to the tangle. The complication is due to mixedness of the bipartite state. This is exactly the point of the entanglement–mixedness relation.

In view of the above discussions, we separate the l.h.s. of Equation (39) into two parts. One part is the tangle τ(ρ) measuring the entanglement, and the other part we identify as the square of the separable uncertainty $B^{2}\left(\rho \right)$. Thus, we have the *generalized entanglement–mixedness relation* for general bipartite systems in mixed states:

(40) $$\left(\tau +B^{2}\right)/\bar{\nu_{k}^{2}}=\tilde{M_{k}^{2}},$$

which is an alternative form of $\tau +B^{2}=\bar{\nu_{k}^{2}M_{k}^{2}}$. The bar notation introduced before represents the equal-weight average, e.g., $\bar{\nu_{k}^{2}}=\sum_{k=1}^{2}\nu_{k}^{2}/2$. The tilde notation (not to be confused with the universal state inversion) introduced here represents the (possibly) unequal-weight average, e.g., $\tilde{M_{k}^{2}}=\sum_{k=1}^{2}w_{k}M_{k}^{2}$, where the weight is given by $w_{k}=\nu_{k}^{2}/\left(\nu_{1}^{2}+\nu_{2}^{2}\right)$. The tangle τ(ρ) is defined by Equation (34), and the separable uncertainty has the expression

(41) $$B\left(\rho \right)=\sqrt{\mathrm{tr}\left(\rho \tilde{\rho }\right)+\nu^{2}M^{2}\left(\rho \right)/2-\tau \left(\rho \right)}=\sqrt{\bar{\nu_{k}^{2}M_{k}^{2}}-\tau \left(\rho \right)}.$$

The separable uncertainty B(ρ) measures the ignorance in the states of the individual subsystems due to the mixedness of the bipartite state instead of the entanglement between the two subsystems. B(ρ) is in the range $\left[0,\sqrt{2-1/n_{1}-1/n_{2}}\right]$, where the minimum is achieved when the bipartite system is in a pure state and the maximum is reached when the bipartite state is the completely mixed state ρ=I/n. In the special case when the bipartite system is in a pure state, we have B=0 and $\tau =C^{2}$, and the average notations (bars and tildes) can be dropped due to the property $\nu_{1}M_{1}=\nu_{2}M_{2}$. Then, we recover $C^{2}\left(\Psi \right)=\nu_{k}^{2}M_{k}^{2}$ or $C\left(\Psi \right)=\nu_{k}M_{k}$ for pure states.

For rank-2 mixed states of bipartite systems, described by density operators with no more than two nonzero eigenvalues, the tangle τ(ρ) has the following expression in our notations [31]:

(42) $$\tau \left(\rho \right)=\mathrm{tr}\left(\rho \tilde{\rho }\right)+\lambda_{\min}\;\nu^{2}M{\left(\rho \right)}^{2},$$

where $\lambda_{\min}$ is the smallest eigenvalue of a certain 3×3 real symmetric matrix that will be specified later. Combining the above expression with Equation (41), we obtain the more explicit form of the separable uncertainty for these rank-2 mixed states:

(43) $$B\left(\rho \right)=\sqrt{\frac{1}{2}-\lambda_{\min}}\;\nu M\left(\rho \right),$$

which is directly connected to the mixedness of the bipartite state. (Note that $\lambda_{\min}$ is invariant under local unitary transformations and so is B(ρ).) This is also an indication that the tangle τ(ρ) is a suitable (simple and powerful) entanglement measure in the formulation of the entanglement–mixedness relation. To what extent the relation in Equation (43) can be expanded to include more general states will be a subject of future investigation.

#### 2.3.2. Generalized Local-Nonlocal Complementarity for Mixed States

Combining the entanglement–mixedness relation in Equation (40) and the purity–mixedness complementarity relation in Equation (19), $Q_{k}^{2}+M_{k}^{2}=1$, we obtain the *local-nonlocal complementarity relation* for general bipartite systems in mixed states:

(44) $$\tilde{Q_{k}^{2}}+\left(\tau +B^{2}\right)/\bar{\nu_{k}^{2}}=1,$$

where the bar and tilde notations have the same meanings as before. This equation relates in a complementary way the local (single-partite) quantum properties characterized by the quantum purity $Q_{k}$, the nonlocal (bipartite) quantum property characterized by the tangle τ measuring the entanglement between the two subsystems, as well as the separable uncertainty B quantifying the ignorance. When the bipartite system is in a pure state, B=0, $\tau =C^{2}$, and the average notations can be dropped. As a result, the above equation reduces to $Q_{k}^{2}+C^{2}/\nu_{k}^{2}=1$ in Equation (26), which represents a trade-off between the local quantum property (purity) and the nonlocal quantum property (entanglement).

#### 2.3.3. Generalized Local Complementarity

The local complementarity relation

(45) $$P_{k}^{2}+V_{k}^{2}=Q_{k}^{2}$$

is not affected by whether the bipartite system is in a pure or mixed state, as it is a local (single-partite) relation entirely determined by the subsystem density matrix. The same is true for the purity–mixedness complementarity relation

(46) $$Q_{k}^{2}+M_{k}^{2}=1.$$

The combination of these two equations also leads to the wave–particle-mixedness (WPM) complementarity relation

(47) $$P_{k}^{2}+V_{k}^{2}+M_{k}^{2}=1,$$

which, with $M_{k}^{2}$ in the form of $S_{L}$, was noted in Reference [33] in a remark. Equations (45)–(47) are all single-partite (local) complementarity relations. They actually apply to a general *n*-dimensional system by dropping the subsystem index *k*.

#### 2.3.4. Generalized WPEI Complementarity for Mixed States

Combining the local-nonlocal complementarity in Equation (44) and the local complementarity in Equation (45), we finally obtain the *generalized WPEI complementarity relation* for general bipartite systems in mixed states:

(48) $$\tilde{P_{k}^{2}}+\tilde{V_{k}^{2}}+\left(\tau +B^{2}\right)/\bar{\nu_{k}^{2}}=1.$$

This relation connects four complementing aspects in a tight equality, namely, the predictability of the subsystems characterizing their particle properties, the visibility of the subsystems quantifying their wave properties, the tangle measuring the entanglement between the two subsystems, and the separable uncertainty representing the ignorance in the individual subsystem states associated with the mixedness of the bipartite state. In the special case when the bipartite system is in a pure state, we have B=0, $\tau =C^{2}$, and the bar and tilde notations can be removed. We then recover the WPE complementarity relation for bipartite systems in pure states in Equation (28): $P_{k}^{2}+V_{k}^{2}+C^{2}/\nu_{k}^{2}=1$.

#### 2.3.5. Summary

The central result in Section 2.3 is the generalized WPEI complementarity relation for general bipartite systems in mixed states:

(49) $$\tilde{P_{k}^{2}}+\tilde{V_{k}^{2}}+\left(\tau +B^{2}\right)/\bar{\nu_{k}^{2}}=1,$$

which is obtained by combining the local-nonlocal complementarity relation

(50) $$\tilde{Q_{k}^{2}}+\left(\tau +B^{2}\right)/\bar{\nu_{k}^{2}}=1$$

and the local complementarity relation

(51) $$P_{k}^{2}+V_{k}^{2}=Q_{k}^{2}.$$

The local-nonlocal complementarity relation is obtained from the entanglement–mixedness relation

(52) $$\left(\tau +B^{2}\right)/\bar{\nu_{k}^{2}}=\tilde{M_{k}^{2}}$$

and the purity–mixedness complementarity relation

(53) $$Q_{k}^{2}+M_{k}^{2}=1.$$

In addition, the combination of Equations (51) and (53) leads to the WPM complementarity relation

(54) $$P_{k}^{2}+V_{k}^{2}+M_{k}^{2}=1.$$

The tangle τ measuring the entanglement is defined in Equation (35). The separable uncertainty B is given by Equation (41). For rank-2 mixed states, τ and B have the more explicit forms in Equations (42) and (43), respectively. The expressions of other quantities have been given in Equations (29)–(32). The bar notation represents the equal-weight average, e.g., $\bar{\nu_{k}^{2}}=\sum_{k=1}^{2}\nu_{k}^{2}/2$, while the tilde notation represents the (possibly) unequal-weight average, e.g., $\tilde{P_{k}^{2}}=\sum_{k=1}^{2}w_{k}P_{k}^{2}$ with $w_{k}=\nu_{k}^{2}/\left(\nu_{1}^{2}+\nu_{2}^{2}\right)$ and $\nu_{k}=\sqrt{2\left(n_{k}-1\right)/n_{k}}$. In the special case of a pure state of the bipartite system, B=0, $\tau =C^{2}$, and the average notations (bars and tildes) can be dropped. The results of the WPE complementarity in Section 2.2 for pure states can then be recovered. The various relations in Equations (49)–(54) may be collectively referred to as the WPEI complementarity relations, while the more specific usage of the term only refers to the relation in Equation (49).

On the formal level, the WPEI complementarity relation for general bipartite systems, $\tilde{P_{k}^{2}}$ + $\tilde{V_{k}^{2}}$ + (τ + $B^{2})/\bar{\nu_{k}^{2}}$ = 1, differs from that for two-qubit systems, $\bar{P_{k}^{2}}+\bar{V_{k}^{2}}+C^{2}+B^{2}=1$, in the following two major aspects.

- One difference is that the equal-weight averages of the normalized single-partite properties (the predictability and the visibility) are replaced by the (possibly) unequal-weight averages. The relative weights depend on the dimensions of the two subsystems. Thus, the proportions of contribution of the two subsystems to the WPEI complementarity relation are different if they have different dimensions. This point is relevant, for instance, in the case when one subsystem represents a small system under study, while the other subsystem represents the measurement device with a large dimension (e.g., the which-way detector in the multi-beam interference experiment).
- The other difference is that the squared concurrence $C^{2}$ is replaced by the tangle τ. For two-qubit systems, these two measures of entanglement are the same. However, they differ for mixed states of general bipartite systems (and coincide only for pure states in general). We have argued, with the support of results in rank-2 states that the tangle, instead of the squared concurrence, is the more suitable (simple and powerful) entanglement measure to use in the formulation of the WPEI complementarity relation.

#### 2.3.6. Schematic Representation of the WPEI Complementarity Relations

**Figure 3:**
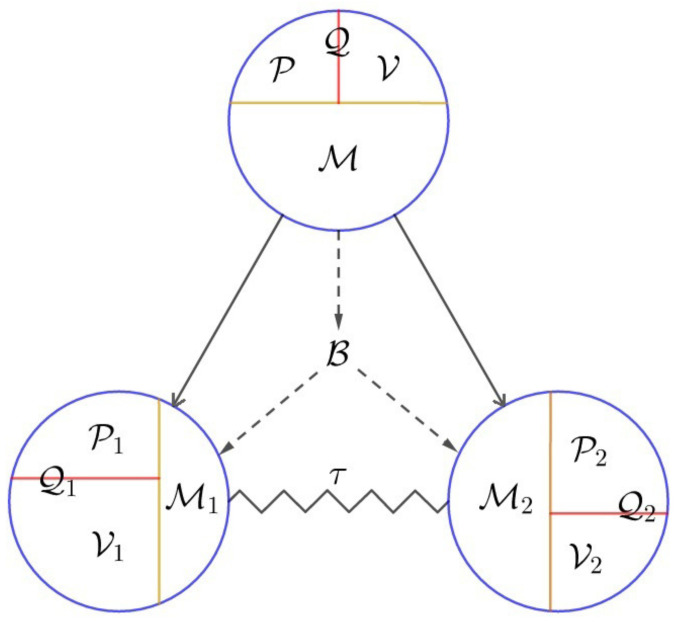
A schematic representation of the WPEI complementarity relations in general bipartite systems.

The WPEI complementarity relations in Equations (49)–(54) can be schematically represented by Figure 3. The top circle represents the bipartite system, which consists of two subsystems indicated by the two circles at the bottom, connected to the top circle by two solid arrows. Each circle is divided into three parts, corresponding to the predictability P, the visibility V, and the mixedness M, respectively. This is a reflection of the WPM complementarity in Equation (54). The two parts associated with the predictability P and the visibility V are combined into one part denoted by the purity Q, a representation of the local complementarity in Equation (51). Each circle can also be regarded as consisting of two parts, associated with the purity Q and the mixedness M, respectively, a reflection of the purity–mixedness complementarity in Equation (53). Since the purity Q characterizes the quantum nature of the system while the mixedness M reflects the missing of quantum information, one may imagine that the part corresponding to the purity Q is full while the part associated with the mixedness M is empty. The wiggling line connecting the two bottom circles symbolizes the entanglement between the two subsystems measured by the tangle τ. The entanglement between the two subsystems contributes to the mixedness of the states of the two individual subsystems. This is why the two ends of the wiggling line are attached to the mixedness regions of the two circles. The wiggling line is solid, reflecting the fact that entanglement indicates the *presence* of a bipartite quantum property. In addition, the mixedness of the states of the two individual subsystems is also contributed by the separable uncertainty B, which originates from the mixedness of the bipartite state. This fact is represented by the three dashed arrows around the separable uncertainty B. The arrows are dashed as the mixedness indicates the *missing* of quantum information. The solid wiggling line and the three dashed arrows connected to the mixedness regions of the three circles are a representation of the entanglement–mixedness relation in Equation (52). The stronger the tangle τ and the separable uncertainty B are, the more mixed the states of the individual subsystems are (larger regions of mixedness), and thus the less pure they are (smaller regions of purity), a manifestation of the local-nonlocal complementarity in Equation (50). Further noting that the purity region consists of the predictability region and the visibility region, we also have the representation of the WPEI complementarity in Equation (49). Therefore, Figure 3 has essentially captured all the complementarity relations in Equations (49)–(54).

### 2.4. WPEI Complementarity for Hierarchical Bipartite Systems

**Figure 4:**
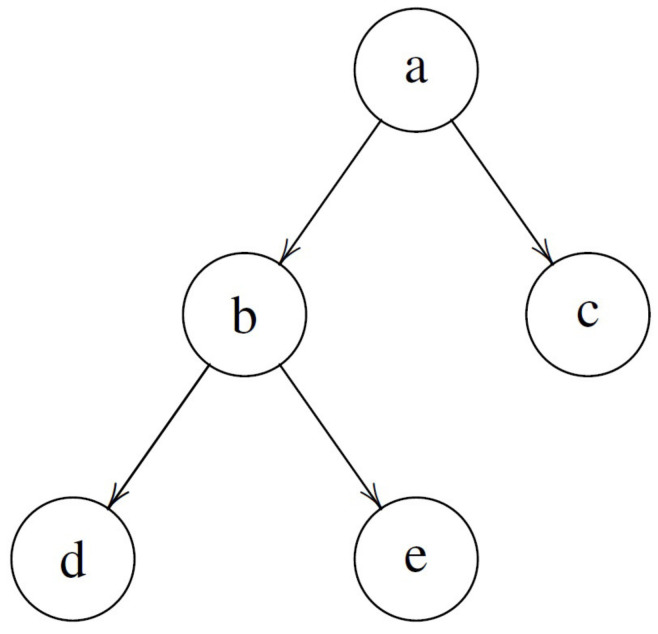
A schematic representation of the structure of a hierarchical bipartite system as a full binary tree.

The WPEI complementarity for general bipartite systems in a general state (pure or mixed) in the form of equalities allows it to be readily applied to a more general class of systems, which we refer to as hierarchical bipartite systems. Hierarchical bipartite systems are bipartite systems consisting of bipartite systems, so that the entire system and all its subsystems at different levels are organized in a hierarchical structure in the form of a binary tree. More precisely, the organization of the system has the structure of a full binary tree, with each subsystem (node) having either no subsystem (no children) or two subsystems (two children). The root node of the binary tree represents the whole system, while other nodes represent subsystems at different levels. A schematic representation of a hierarchical bipartite system is shown in Figure 4. In this example, the whole system *a* is a bipartite system, consisting of subsystem *b* and subsystem *c*. Subsystem *b* is yet another bipartite system, consisting of subsystem *d* and subsystem *e*.

Hierarchical bipartite systems are widely encountered. A hierarchical bipartite system can be simply constructed by dividing a system into two parts successively. For a given system, there are different ways to divide the system and its subsystems into two parts. Therefore, different hierarchical bipartite systems can be constructed from the same whole system, depending on how the system and its subsystems are divided into two parts. For instance, for a system consisting of three qubits, there are three different ways to divide the three qubits into two parts (one part containing one qubit and the other containing two), corresponding to three different hierarchical bipartite systems with two levels in the binary tree. If the subsystem consisting of two qubits is further divided into two parts, each containing one qubit, and then we obtain another three different hierarchical bipartite systems with three levels in the binary tree, which have the same structure as that shown in Figure 4.

Since the WPEI complementarity relations in Equations (49)–(54), schematically represented by Figure 3, apply to general multi-dimensional bipartite systems in pure or mixed states, it is a simple matter of realization that they also apply to each bipartite subsystem in the hierarchical bipartite system. Roughly speaking, the structure in Figure 3 is Λ-shaped (the basic structure in a full binary tree), but with more specific modifications (e.g., the bottom nodes are connected due to the presence of entanglement). By replacing the Λ-shaped basic structures in the full binary tree of the hierarchical bipartite system (e.g., those in Figure 4) with the more detailed structure in Figure 3, we have the graphical representation of the WPEI complementarity relations for the hierarchical bipartite system. Translating the graphical representation into mathematical formulas using Equations (49)–(54), the mathematical form of the WPEI complementarity relations for the hierarchical bipartite system can also be obtained.

Take the hierarchical bipartite system in Figure 4 for instance. The WPEI complementarity relation for the b-d-e bipartite subsystem reads

(55) $$\tilde{P_{d,e}^{2}}+\tilde{V_{d,e}^{2}}+\left[\tau_{de}+{\left(B_{de}^{b}\right)}^{2}\right]/\bar{\nu_{d,e}^{2}}=1,$$

where we have used the system labels to indicate the various quantities involved. The label “d,e” represents *d* or *e*. The label “de” in the tangle represents *d* and *e* as the entanglement is a bipartite property. The label “${}_{de}^{b}$” in $B_{de}^{b}$ indicates that the separable uncertainty is related to three subsystems, one on the higher level (subsystem *b*) and the other two on the lower level (subsystems *d* and *e*). This reflects the understanding that the separable uncertainty originates from the mixedness of the bipartite state and contributes to the mixedness of the states of the individual subsystems. Similarly, the WPEI complementarity relation for the a-b-c bipartite subsystem reads

(56) $$\tilde{P_{b,c}^{2}}+\tilde{V_{b,c}^{2}}+\left[\tau_{bc}+{\left(B_{bc}^{a}\right)}^{2}\right]/\bar{\nu_{b,c}^{2}}=1.$$

Other complementarity relations in Equations (49)–(54) can also be obtained simply by adding system labels.

As mentioned, different hierarchical bipartite systems can be built from the same whole system. The WPEI complementarity relations apply to all these hierarchical bipartite systems constructed from the same system. The consistency of all these relations may provide a means to investigate, for instance, the multi-partite entanglement, which will be pursued in the future.

We remark that the terms “local” and "nonlocal" used in this article as in the “local-nonlocal complementarity” only have relative meanings in light of the structure of the hierarchical bipartite system. A single-partite property (e.g., the visibility of a subsystem) may represent a local property when considered in relation to the higher-level system, but may represent a nonlocal property when considered in relation to the lower-level subsystems. The connections of the system properties at different levels are worth further investigation.

### 2.5. WPEI Complementarity for Infinite-Dimensional Bipartite Systems

We formally generalize the results obtained so far to infinite-dimensional bipartite systems by taking the limit $n_{k}\to \infty$ (k=1,2). (This setting implies that the representation in which the predictability and the visibility are defined has a discrete basis.) Given that $\nu_{k}=\sqrt{2\left(n_{k}-1\right)/n_{k}}$, in the limit $n_{k}\to \infty$, we have $\nu_{k}=\sqrt{2}$, so that $\bar{\nu_{k}^{2}}=2$ and $w_{k}=\nu_{k}^{2}/\left(\nu_{1}^{2}+\nu_{2}^{2}\right)=1/2$. The latter means the unequal-weight averages (the tilde notations) reduce to the equal-weight averages (the bar notations). As a result, for infinite-dimensional bipartite systems, we have the WPEI complementarity relation

(57) $$\bar{P_{k}^{2}}+\bar{V_{k}^{2}}+\frac{1}{2}\left(\tau +B^{2}\right)=1,$$

the local-nonlocal complementarity relation

(58) $$\bar{Q_{k}^{2}}+\frac{1}{2}\left(\tau +B^{2}\right)=1,$$

the entanglement–mixedness relation

(59) $$\frac{1}{2}\left(\tau +B^{2}\right)=\bar{M_{k}^{2}},$$

the local complementarity relation

(60) $$P_{k}^{2}+V_{k}^{2}=Q_{k}^{2},$$

the purity–mixedness complementarity relation

(61) $$Q_{k}^{2}+M_{k}^{2}=1,$$

and the WPM complementarity relation

(62) $$P_{k}^{2}+V_{k}^{2}+M_{k}^{2}=1.$$

The expressions of the relevant quantities read

(63) $$\begin{array}{ccc} P_{k} & = & \sqrt{\sum_{i}{\left(\rho_{ii}^{k}\right)}^{2}}, \end{array}$$

(64) $$\begin{array}{ccc} V_{k} & = & \sqrt{\sum_{i\neq j}{\left|\rho_{ij}^{k}\right|}^{2}}, \end{array}$$

(65) $$\begin{array}{ccc} Q_{k} & = & \sqrt{\mathrm{tr}(\rho_{k}^{2})}, \end{array}$$

(66) $$\begin{array}{ccc} M_{k} & = & \sqrt{1-\mathrm{tr}(\rho_{k}^{2})}, \end{array}$$

(67) $$\begin{array}{ccc} C(\Psi ) & = & \sqrt{2[1-\mathrm{tr}\left(\rho_{k}^{2}\right)]}, \end{array}$$

(68) $$\begin{array}{ccc} B & = & \sqrt{\mathrm{tr}\left(\rho \tilde{\rho }\right)+M^{2}\left(\rho \right)-\tau \left(\rho \right)}=\sqrt{2\bar{M_{k}^{2}}-\tau \left(\rho \right)}, \end{array}$$

and the tangle τ(ρ) is still defined by the convex roof extension of the pure-state measure $\tau \left(\Psi \right)=C^{2}\left(\Psi \right)$. For the infinite series in $P_{k}$ and $V_{k}$ to converge, the reduced density operator $\rho_{k}$ need to be normalizable, e.g., $\mathrm{tr}(\rho_{k})=1$, implying that $\rho_{k}$ is a trace class operator.

When the infinite-dimensional bipartite system is in a pure state, B=0, $\tau =C^{2}$ and the bar notations can be removed. Then, we have the WPE complementarity relation

(69) $$P_{k}^{2}+V_{k}^{2}+\frac{1}{2}C^{2}=1,$$

the local-nonlocal complementarity relation

(70) $$Q_{k}^{2}+\frac{1}{2}C^{2}=1,$$

the local complementarity relation

(71) $$P_{k}^{2}+V_{k}^{2}=Q_{k}^{2},$$

and the entanglement–mixedness relation

(72) $$C=\sqrt{2}M_{k}.$$

Equations (60) and (61) are implied by the above equations. If needed, $C/\sqrt{2}$ may be introduced as a normalized concurrence to bring the formulas into a more elegant form.

## 3. Examples

To demonstrate the general formulation obtained in the previous sections, we study two more specific examples. The first example is the simplest nontrivial bipartite system with unequal dimensions, consisting of a qubit (two-state system) and a qutrit (three-state system). In this example, we consider rank-2 mixed states which allow explicit calculations of the tangle and the separable uncertainty. The second example is an infinite-dimensional bipartite system consisting of two quantons in pure entangled coherent states.

### 3.1. WPEI Complementarity in Rank-2 Mixed States of a Qubit–Qutrit System

Consider a qubit–qutrit system with the computational basis {|00〉,|01〉,|02〉,|10〉,|11〉,|12〉}. We study its rank-2 states described by the following density operator:

(73) $$\rho =p|v_{1}\rangle \langle v_{1}\left|+\left(1-p\right)\right|v_{2}\rangle \langle v_{2}|,$$

where $|v_{1}\rangle$ and $|v_{2}\rangle$ are eigenstates of ρ defined as

(74) $$|v_{1}\rangle =\frac{1}{\sqrt{2}}(|00\rangle +\left|11\rangle ),\;\right|v_{2}\rangle =\frac{1}{\sqrt{3}}(|01\rangle +|02\rangle +|12\rangle ),$$

which are both entangled states. By construction, ρ has at most two nonzero eigenvalues *p* and 1−p. When p=0 or 1, it reduces to the pure states $|v_{2}\rangle$ and $|v_{1}\rangle$, respectively. Otherwise, it represents a mixed state.

The reduced density operators of the qubit and the qutrit are obtained by direct calculation, which yields

(75) $$\rho_{1}={\mathrm{tr}}_{2}\left(\rho \right)=\frac{1}{6}\left(4-p\right)|0\rangle \langle 0|+\frac{1}{6}\left(2+p\right)|1\rangle \langle 1|+\frac{1}{3}\left(1-p\right)(|0\rangle \langle 1|+|1\rangle \langle 0|),$$

(76) $$\rho_{2}={\mathrm{tr}}_{1}\left(\rho \right)=\frac{p}{2}\left|0\rangle \langle 0\right|+\frac{1}{6}\left(2+p\right)|1\rangle \langle 1|+\frac{2}{3}\left(1-p\right)|2\rangle \langle 2|+\frac{1}{3}\left(1-p\right)(|1\rangle \langle 2|+|2\rangle \langle 1|).$$

#### 3.1.1. Local Complementarity Relations within the Qubit

In the computational basis, the density operator $\rho_{1}$ is represented by the density matrix with the elements

(77) $$\rho_{00}^{\left(1\right)}=\frac{1}{6}\left(4-p\right),\;\rho_{11}^{\left(1\right)}=\frac{1}{6}\left(2+p\right),\;\rho_{01}^{\left(1\right)}=\rho_{10}^{\left(1\right)}=\frac{1}{3}\left(1-p\right).$$

Applying Equations (29)–(32) to this case, we obtain the predictability

(78) $$P_{1}=\sqrt{2\left[{\left(\rho_{00}^{\left(1\right)}\right)}^{2}+{\left(\rho_{11}^{\left(1\right)}\right)}^{2}-\frac{1}{2}\right]}=\frac{1}{3}\left(1-p\right),$$

the visibility

(79) $$V_{1}=2\left|\rho_{01}^{\left(1\right)}\right|=\frac{2}{3}\left(1-p\right),$$

the purity

(80) $$Q_{1}=\sqrt{2\mathrm{tr}(\rho_{1}^{2})-1}=\frac{\sqrt{5}}{3}\left(1-p\right),$$

and the mixedness

(81) $$M_{1}=\sqrt{2[1-\mathrm{tr}\left(\rho_{1}^{2}\right)]}=\frac{1}{3}\sqrt{4+10p-5p^{2}}.$$

It is easy to check the local complementarity $P_{1}^{2}+V_{1}^{2}=Q_{1}^{2}$, the purity–mixedness complementarity $Q_{1}^{2}+M_{1}^{2}=1$, and the WPM complementarity $P_{1}^{2}+V_{1}^{2}+M_{1}^{2}=1$ for this qubit.

Notice that both the predictability $P_{1}$ and the visibility $V_{1}$ decrease as *p* increases, indicating that the particle property and the wave property are not acting in a complementary way in this case. This is because the purity $Q_{1}$ is not held constant, but also decreases with *p* in this case. When the purity Q varies, it is possible that the predictability (particle aspect) and the visibility (wave aspect) may both increase or decrease.

#### 3.1.2. Local Complementarity Relations within the Qutrit

In the computational basis, the density operator $\rho_{2}$ is represented by the density matrix with the following nonzero elements:

(82) $$\rho_{00}^{\left(2\right)}=\frac{p}{2},\;\rho_{11}^{\left(2\right)}=\frac{1}{6}\left(2+p\right),\;\rho_{22}^{\left(2\right)}=\frac{2}{3}\left(1-p\right),\;\rho_{12}^{\left(2\right)}=\rho_{21}^{\left(2\right)}=\frac{1}{3}\left(1-p\right).$$

With the help of Equations (29)–(32) and some algebra, we get the predictability

(83) $$P_{2}=\sqrt{\frac{3}{2}\left[\sum_{i=0}^{2}{\left(\rho_{ii}^{\left(2\right)}\right)}^{2}-\frac{1}{3}\right]}=\frac{\sqrt{4-14p+13p^{2}}}{2\sqrt{3}},$$

the visibility

(84) $$V_{2}=\sqrt{3}\left|\rho_{12}^{\left(2\right)}\right|=\frac{1}{\sqrt{3}}\left(1-p\right),$$

the purity

(85) $$Q_{2}=\sqrt{\frac{3}{2}\mathrm{tr}\left(\rho_{2}^{2}\right)-\frac{1}{2}}=\frac{\sqrt{8-22p+17p^{2}}}{2\sqrt{3}},$$

and the mixedness

(86) $$M_{2}=\sqrt{\frac{3}{2}\left[1-\mathrm{tr}\left(\rho_{2}^{2}\right)\right]}=\frac{\sqrt{4+22p-17p^{2}}}{2\sqrt{3}}.$$

The local complementarity $P_{2}^{2}+V_{2}^{2}=Q_{2}^{2}$, the purity–mixedness complementarity $Q_{2}^{2}+M_{2}^{2}=1$, and the WPM complementarity $P_{2}^{2}+V_{2}^{2}+M_{2}^{2}=1$ are also easily verified for this qutrit.

#### 3.1.3. Tangle and Separate Uncertainty

Since the density operator ρ in this case represents rank-2 states, we can use Equation (42) to calculate the tangle [31], which is reproduced below for the reader’s convenience:

(87) $$\tau \left(\rho \right)=\mathrm{tr}\left(\rho \tilde{\rho }\right)+\lambda_{\min}\;\nu^{2}M{\left(\rho \right)}^{2},$$

where $\tilde{\rho }$ is the universal state inversion of ρ, and $\lambda_{\min}$ is the smallest eigenvalue of a certain 3×3 real symmetric matrix. The universal state inversion for a general linear operator *K* (not necessarily Hermitian) is defined as follows [31]:

(88) $$\tilde{K}=\mathrm{tr}\left(K^{†}\right)I-K_{1}^{†}⊗I_{2}-I_{1}⊗K_{2}^{†}+K^{†},$$

where $K_{1}={\mathrm{tr}}_{2}\left(K\right)$ and $K_{2}={\mathrm{tr}}_{1}\left(K\right)$.

The term $\mathrm{tr}(\rho \tilde{\rho })$ can be calculated using the above definition of $\tilde{\rho }$, or more conveniently using the property derived from it (see Equation (38)):

(89) $$\mathrm{tr}\left(\rho \tilde{\rho }\right)=1-\mathrm{tr}\left(\rho_{1}^{2}\right)-\mathrm{tr}\left(\rho_{2}^{2}\right)+\mathrm{tr}\left(\rho^{2}\right)=\frac{1}{9}\left(4-2p+7p^{2}\right).$$

The mixedness of the qubit–qutrit state is given by

(90) $$M\left(\rho \right)=\sqrt{\frac{6}{5}\left[1-\mathrm{tr}\left(\rho^{2}\right)\right]}=\sqrt{\frac{12}{5}p\left(1-p\right)},$$

and $\nu =\sqrt{2(n-1)/n}=\sqrt{5/3}$.

Then, we still need to calculate $\lambda_{\min}$, the minimum eigenvalue of a real symmetric 3×3 matrix. This matrix was denoted by *M* in Reference [31]. To avoid confusion with the mixedness, we shall denote it by *G*. Before calculating $\lambda_{\min}$, a couple of typos or minor errors in the expression of the matrix *G* (the matrix *M* defined by Equation (18) in [31]) need to be corrected. The corrected expression is given by

(91) $$G_{jk}=\frac{1}{4}\sum_{\alpha }\mathrm{tr}\left(\sigma_{j}^{*}\zeta_{\alpha }^{†}\sigma_{k}{\left(\zeta_{\alpha }^{†}\right)}^{*}\right),$$

where the star represents a complex conjugate, the dagger represents Hermitian conjugate, and $\sigma_{i}$ (i=1,2,3) are Pauli matrices. $\zeta_{\alpha }$ is a 2×2 matrix with its elements defined as

(92) $$\zeta_{ij}^{\alpha }=\langle v_{i}|{\vec{\theta }}_{\alpha }|v_{j}\rangle =\langle v_{i}|{\tilde{v}}_{j}^{\alpha }\rangle ,$$

where $|{\tilde{v}}_{j}^{\alpha }\rangle \equiv {\vec{\theta }}_{\alpha }|v_{j}\rangle$, $\theta_{\alpha }$ is an anti-linear operator labeled by α, and the arrow over it indicates the direction it acts on (to the right in this case). Compared with Equation (18) in [31], there are two differences in Equation (91). One difference is that there is an additional factor 1/4. The second difference is that the matrix $\zeta_{\alpha }^{†}$ has replaced $\zeta_{\alpha }$. (Equivalently, if $\zeta_{\alpha }$ instead of $\zeta_{\alpha }^{†}$ is used in Equation (91), then the anti-linear operator $\theta_{\alpha }$ in Equation (92) should act to the left instead of to the right.) The first difference is due to a typo, as the component-wise expression given in Equation (12) in [31] does have the factor 1/4. We have carefully examined the derivation of $G_{jk}$ ($M_{jk}$ in [31]) and also compared with a previous work [32] that the derivation was based on. $\zeta_{\alpha }^{†}$ instead of $\zeta_{\alpha }$ should be used in the correct expression. The form of $G_{jk}$ in Equation (91) containing the anti-linear operators is not convenient to use. An alternative expression can be obtained following [31] (using the corrected expression):

(93) $$G_{ij}=\frac{1}{4}\sum_{klmn}T_{mknl}{\left[\sigma_{i}^{*}\right]}_{kl}{\left[\sigma_{j}\right]}_{mn},$$

where $T_{mknl}=\mathrm{tr}\left(\gamma_{mk}{\tilde{\gamma }}_{nl}\right)$, $\gamma_{mk}=|v_{m}\rangle \langle v_{k}|$, and ${\tilde{\gamma }}_{nl}=\sum_{\alpha }|{\tilde{v}}_{n}^{\alpha }\rangle \langle {\tilde{v}}_{l}^{\alpha }|$. Note that ${\tilde{\gamma }}_{nl}$ is the universal state inversion of $\gamma_{nl}$, which can be calculated using Equation (88) without referring to the anti-linear operators.

After some tedious algebra, we obtain

(94) $$\begin{array}{cc} T_{1111}=1, & \;T_{1122}=T_{1212}=T_{2121}=T_{2211}=\frac{1}{3},\\ T_{2222}=\frac{4}{9}, & \;T_{1222}=T_{2122}=T_{2212}=T_{2221}=-\frac{2}{3\sqrt{6}}, \end{array}$$

and the rest of the components are zero. Then, the expression in Equation (93) yields

(95) $$G=\left[\begin{array}{ccc} \frac{1}{6} & 0 & \frac{1}{3\sqrt{6}}\\ 0 & \frac{1}{6} & 0\\ \frac{1}{3\sqrt{6}} & 0 & \frac{7}{36} \end{array}\right].$$

(We note that the component-wise expressions in Equation (12) in [31] give the same matrix above in this case. However, we are not sure whether this is generally true.) The eigenvalues of the matrix *G* are given by $\lambda =1/6,(13\pm \sqrt{97})/72$, so that the smallest eigenvalue is

(96) $$\lambda_{\min}=\frac{1}{72}\left(13-\sqrt{97}\right).$$

Combining Equations (87), (89), (90) and (96), we finally obtain the tangle quantifying the entanglement between the qubit and qutrit:

(97) $$\tau \left(\rho \right)=\mathrm{tr}\left(\rho \tilde{\rho }\right)+\lambda_{\min}\;\nu^{2}M{\left(\rho \right)}^{2}=\frac{1}{9}\left(4-2p+7p^{2}\right)+\frac{1}{18}\left(13-\sqrt{97}\right)p\left(1-p\right).$$

The separate uncertainty can also be found in this case using Equation (43):

(98) $$B\left(\rho \right)=\sqrt{\frac{1}{2}-\lambda_{\min}}\;\nu M\left(\rho \right)=\sqrt{\frac{1}{18}\left(23+\sqrt{97}\right)p\left(1-p\right)}.$$

#### 3.1.4. Entanglement–Mixedness Relation

We verify the entanglement–mixedness relation

(99) $$\left(\tau +B^{2}\right)/\bar{\nu_{k}^{2}}=\tilde{M_{k}^{2}}.$$

Note that $\nu_{1}=1$ and $\nu_{2}=2/\sqrt{3}$, so that $\bar{\nu_{k}^{2}}=7/6$ and the weights in the unequal-weight average read $w_{1}=3/7$, $w_{2}=4/7$.

According to the expressions of τ and B in Equations (97) and (98), we have

(100) $$\left(\tau +B^{2}\right)/\bar{\nu_{k}^{2}}=\frac{2}{21}\left(4+16p-11p^{2}\right).$$

On the other hand, with the expressions of $M_{1}$ and $M_{2}$ given in Equations (81) and (86), we obtain the average squared mixedness

(101) $$\tilde{M_{k}^{2}}=\frac{3}{7}M_{1}^{2}+\frac{4}{7}M_{2}^{2}=\frac{2}{21}\left(4+16p-11p^{2}\right).$$

Hence, the entanglement–mixedness relation is verified.

As a consistency check, consider the special cases p=0 and p=1, in which the bipartite state is pure, corresponding to $|v_{2}\rangle$ and $|v_{1}\rangle$, respectively. In these two cases, the separate uncertainty vanishes as expected. In addition, the tangles, according to Equation (97), are given by $\tau (|v_{2}\rangle )=4/9$ and $\tau (|v_{1}\rangle )=1$. Equivalently, the concurrences are given by $C(|v_{2}\rangle )=2/3$ and $C(|v_{1}\rangle )=1$, according to the relation $\tau \left(\Psi \right)=C^{2}\left(\Psi \right)$. On the other hand, the subsystems in this case have the following mixedness: $M_{1}(|v_{2}\rangle )=2/3$, $M_{2}(|v_{2}\rangle )=1/\sqrt{3}$, $M_{1}(|v_{1}\rangle )=1$, $M_{2}(|v_{1}\rangle )=\sqrt{3}/2$. With $\nu_{1}=1$ and $\nu_{2}=2/\sqrt{3}$, it is easy to see that these results agree with the entanglement–mixedness relation for pure states $C\left(\Psi \right)=\nu_{k}M_{k}$.

#### 3.1.5. Local-Nonlocal Complementarity

The local-nonlocal complementarity for mixed states reads

(102) $$\tilde{Q_{k}^{2}}+\left(\tau +B^{2}\right)/\bar{\nu_{k}^{2}}=1.$$

Direct calculation yields the average squared purity

(103) $$\tilde{Q_{k}^{2}}=\frac{3}{7}Q_{1}^{2}+\frac{4}{7}Q_{2}^{2}=\frac{1}{21}\left(13-32p+22p^{2}\right),$$

where we have used $Q_{1}$ and $Q_{2}$ in Equations (80) and (85). With $\left(\tau +B^{2}\right)/\bar{\nu_{k}^{2}}$ in Equation (100), the local-nonlocal complementarity is easily shown to hold true in this case.

#### 3.1.6. WPEI Complementarity

The WPEI complementarity relation has the form

(104) $$\tilde{P_{k}^{2}}+\tilde{V_{k}^{2}}+\left(\tau +B^{2}\right)/\bar{\nu_{k}^{2}}=1.$$

The average squared predictability is found to be

(105) $$\tilde{P_{k}^{2}}=\frac{3}{7}P_{1}^{2}+\frac{4}{7}P_{2}^{2}=\frac{1}{21}\left(5-16p+14p^{2}\right),$$

where we have used the expressions of $P_{1}$ and $P_{2}$ in Equations (78) and (83), respectively. Similarly, the average squared visibility is calculated as

(106) $$\tilde{V_{k}^{2}}=\frac{3}{7}V_{1}^{2}+\frac{4}{7}V_{2}^{2}=\frac{8}{21}{\left(1-p\right)}^{2},$$

with $V_{1}$ and $V_{2}$ given in Equations (79) and (84). Together with $\left(\tau +B^{2}\right)/\bar{\nu_{k}^{2}}$ in Equation (100), simple algebra verifies the WPEI complementarity relation in Equation (104).

We have thus validated all the complementarity relations in Equations (49)–(54) and obtained explicit expressions of the various quantities involved for this qubit–qutrit system with rank-2 states. In the following, we study another example with infinite dimensions that can also be handled analytically.

### 3.2. WPE Complementarity Relations in Entangled Coherent States of a Pair of Quantons

We consider the infinite-dimensional bipartite system consisting of two quantons in a pure entangled coherent state [34]

(107) $$|\Psi \rangle =\alpha (|z_{1}z_{2}\rangle +|z_{2}z_{1}\rangle ),$$

where $|z_{1}z_{2}\rangle =|z_{1}\rangle ⊗|z_{2}\rangle$ and $|z_{i}\rangle$ (i=1,2) are single-mode bosonic coherent states defined as $|z\rangle =e^{za^{†}-z^{*}a}|0\rangle$. The pre-factor α, which normalizes the entangled coherent state, has the expression

(108) $$\alpha =\frac{1}{\sqrt{2(1+|\langle z_{1}|z_{2}\rangle {|}^{2})}},$$

where

(109) $$\langle z_{1}|z_{2}\rangle =e^{-\frac{1}{2}\left(\right|z_{1}{|}^{2}+|z_{2}{|}^{2}-2z_{1}^{*}z_{2})}.$$

(Note that $|\langle z_{1}|z_{2}\rangle {|}^{2}=e^{-|z_{1}-z_{2}{|}^{2}}$.)

The density operator for the bipartite system thus reads

(110) $$\rho =\left|\Psi \rangle \langle \Psi \right|=\alpha^{2}(|z_{1}z_{2}\rangle +|z_{2}z_{1}\rangle )(\langle z_{1}z_{2}|+\langle z_{2}z_{1}|).$$

Without loss of generality, we focus on the first quanton, whose reduced density operator is given by

(111) $$\rho_{1}={\mathrm{tr}}_{2}\left(\rho \right)=\alpha^{2}\left(|z_{1}\rangle \langle z_{1}\left|+\right|z_{2}\rangle \langle z_{2}|+\langle z_{1}|z_{2}\rangle |z_{1}\rangle \langle z_{2}|+\langle z_{2}|z_{1}\rangle |z_{2}\rangle \langle z_{1}|\right).$$

#### 3.2.1. Purity

We first calculate the purity Q of quanton 1 (also equal to the purity of quanton 2). According to Equation (65), we have

(112) $$\begin{array}{cc} Q^{2} & =\mathrm{tr}\left({\left(\rho_{1}\right)}^{2}\right)=\alpha^{4}\mathrm{tr}\left[{\left(|z_{1}\rangle \langle z_{1}\left|+\right|z_{2}\rangle \langle z_{2}|+\langle z_{1}|z_{2}\rangle |z_{1}\rangle \langle z_{2}|+\langle z_{2}|z_{1}\rangle |z_{2}\rangle \langle z_{1}|\right)}^{2}\right]\\ \; & =2\alpha^{4}\left(|\langle z_{1}|z_{2}\rangle {|}^{4}+6{\left|\langle z_{1}|z_{2}\rangle \right|}^{2}+1\right)=\frac{|\langle z_{1}|z_{2}\rangle {|}^{4}+6{\left|\langle z_{1}|z_{2}\rangle \right|}^{2}+1}{2(1+|\langle z_{1}|z_{2}\rangle {{|}^{2})}^{2}}. \end{array}$$

Thus, we obtain the purity

(113) $$Q=\frac{\sqrt{|\langle z_{1}|z_{2}\rangle {|}^{4}+6{\left|\langle z_{1}|z_{2}\rangle \right|}^{2}+1}}{\sqrt{2}\left(1+\right|\langle z_{1}|z_{2}\rangle {|}^{2})}.$$

The condition for Q=1 (indicating that the reduced state of the quanton is pure) is given by

(114) $$|\langle z_{1}|z_{2}\rangle {|}^{4}+6|\langle z_{1}|z_{2}\rangle {|}^{2}+1=2(1+|\langle z_{1}|z_{2}\rangle {{|}^{2})}^{2},$$

which yields $|\langle z_{1}|z_{2}\rangle |=1$. Noticing that $|\langle z_{1}|z_{2}\rangle {|}^{2}=e^{-|z_{1}-z_{2}{|}^{2}}$, this condition is equivalent to $|z_{1}-z_{2}|=0$, namely,

(115) $$z_{1}=z_{2}=z.$$

In this special case, it is clear from Equation (107) that the bipartite system is in the pure state |Ψ〉=|zz〉 and the reduced state of each quanton is also pure. Other than the special case $z_{1}=z_{2}=z$, we have Q<1, which means the individual quanton is in a mixed state. According to the expression of Q in Equation (113), the infimum of the purity Q, associated with the least pure state of the individual quanton, is given by

(116) $$Q_{\inf}=\frac{1}{\sqrt{2}},$$

which is asymptotically approached as $|z_{1}-z_{2}|\to \infty$ (i.e., $|\langle z_{1}|z_{2}\rangle |\to 0$).

#### 3.2.2. Concurrence

The concurrence measuring the entanglement between the two quantons, according to Equation (67), is given by

(117) $$C=\sqrt{2[1-\mathrm{tr}\left(\rho_{1}^{2}\right)]}=\sqrt{2(1-Q^{2})}=\frac{1-|\langle z_{1}|z_{2}\rangle {|}^{2}}{1+|\langle z_{1}|z_{2}\rangle {|}^{2}}=\tanh(|z_{1}-z_{2}{|}^{2}/2),$$

which is a monotonically increasing function of $|z_{1}-z_{2}|$. When $z_{1}=z_{2}$, we have C=0, indicating that the quanton is in a separable state with zero entanglement, which is true since |Ψ〉=|zz〉 is a separable pure state. When $z_{1}\neq z_{2}$, we have C>0, indicating that the bipartite system is in an entangled state, which is referred to as the entangled coherent state according to its form of construction. In the limit $|z_{1}-z_{2}|\to \infty$, we have C→1, the supremum of the concurrence (measuring the maximum entanglement that can be asymptotically achieved) for this family of entangled coherent states. (Note that the concurrence C is not normalized and may exceed one in general.)

#### 3.2.3. Local-Nonlocal Complementarity Relation

**Figure 5:**
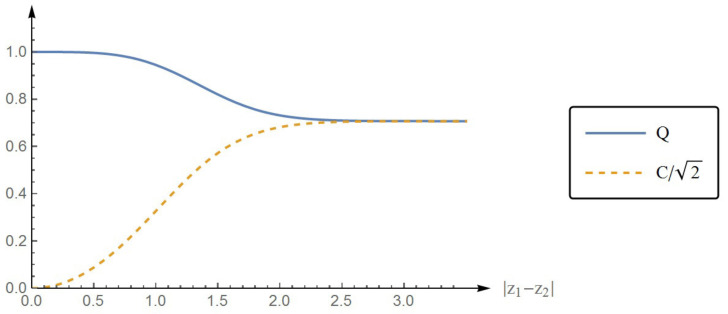
The graphs of the purity Q and the concurrence C (divided by $\sqrt{2}$) as functions of $|z_{1}-z_{2}|$.

The behaviors of the concurrence above mirror those of the purity, as they are connected by the local-nonlocal complementarity relation

(118) $$Q^{2}+\frac{1}{2}C^{2}=1,$$

or $Q^{2}+{\left(C/\sqrt{2}\right)}^{2}=1$. This demonstrates the trade-off between the local property (the purity of the individual quanton) and the nonlocal property (the entanglement between the two quantons). The more entangled the two quantons are, the less pure each quanton is. In Figure 5, we plotted the purity Q and the concurrence C (divided by $\sqrt{2}$) as functions of $|z_{1}-z_{2}|$. The complementarity between Q and C is clear in the figure as can be seen from the anti-correlation of their graphs. In the limit $|z_{1}-z_{2}|\to \infty$, we have $Q_{\inf}=C_{\sup}/\sqrt{2}=1/\sqrt{2}$.

#### 3.2.4. Predictability

The predictability and the visibility are dependent on the local representation. The representation in which we study them in this example is the Fock-state representation spanned by {|n〉}. According to the expression of the reduced density operator $\rho_{1}$ in Equation (111), the density matrix elements in the Fock-state representation are given by

(119) $$\rho_{nm}^{\left(1\right)}=\alpha^{2}\sum_{i,j=1}^{2}\langle z_{i}|z_{j}\rangle \langle n|z_{i}\rangle \langle z_{j}|m\rangle =\alpha^{2}\sum_{i,j=1}^{2}R_{ij}\frac{z_{i}^{n}}{\sqrt{n!}}\frac{z_{j}^{*m}}{\sqrt{m!}},$$

where

(120) $$R_{ij}=e^{-|z_{i}{|}^{2}-{\left|z_{j}\right|}^{2}+z_{i}^{*}z_{j}}.$$

The predictability is only determined by the diagonal elements of the density matrix

(121) $$\rho_{nn}^{\left(1\right)}=\alpha^{2}\sum_{i,j=1}^{2}R_{ij}\frac{\beta_{ij}^{n}}{n!},$$

where we have introduced

(122) $$\beta_{ij}=z_{i}z_{j}^{*}.$$

Then, the predictability is calculated according to Equation (63) as follows:

(123) $$\begin{array}{cc} P^{2} & =\sum_{n=0}^{\infty }{\left(\rho_{nn}^{\left(1\right)}\right)}^{2}=\alpha^{4}\sum_{ijkl}R_{ij}R_{lk}\sum_{n=0}^{\infty }\frac{{\left(\beta_{ij}\beta_{lk}\right)}^{n}}{{\left(n!\right)}^{2}}=\alpha^{4}\sum_{ijkl}R_{ij}R_{lk}\sum_{n=0}^{\infty }\frac{{\left(\beta_{ik}\beta_{lj}\right)}^{n}}{{\left(n!\right)}^{2}}\\ \; & =\alpha^{4}\sum_{ijkl}R_{ij}R_{lk}I_{0}\left(2\sqrt{\beta_{ik}\beta_{lj}}\right), \end{array}$$

where $I_{0}\left(x\right)$ is the modified Bessel function of the first kind with the series expression

(124) $$I_{0}\left(x\right)=\sum_{n=0}^{\infty }\frac{1}{{\left(n!\right)}^{2}}{\left(\frac{x}{2}\right)}^{2n}.$$

In deriving Equation (123), we have also used the property $\beta_{ij}\beta_{lk}=\beta_{ik}\beta_{lj}$ according to the definition in Equation (122), which is to make explicit connection with the visibility discussed later. In addition, the square root (of a complex number) $\sqrt{\beta_{ik}\beta_{lj}}$ is understood as the principal one. Thus, we obtain the expression of the predictability in the Fock-state representation for quanton 1:

(125) $$P=\alpha^{2}\sqrt{\sum_{ijkl}R_{ij}R_{lk}I_{0}\left(2\sqrt{\beta_{ik}\beta_{lj}}\right)},$$

where, more explicitly, we have

(126) $$\begin{array}{ccc} R_{11} & = & e^{-|z_{1}{|}^{2}},\;\beta_{11}={\left|z_{1}\right|}^{2} \end{array}$$

(127) $$\begin{array}{ccc} R_{22} & = & e^{-|z_{2}{|}^{2}},\;\beta_{22}={\left|z_{2}\right|}^{2} \end{array}$$

(128) $$\begin{array}{ccc} R_{12} & = & e^{-|z_{1}{|}^{2}-{\left|z_{2}\right|}^{2}+z_{1}^{*}z_{2}},\;\beta_{12}=z_{1}z_{2}^{*} \end{array}$$

(129) $$\begin{array}{ccc} R_{21} & = & e^{-|z_{1}{|}^{2}-{\left|z_{2}\right|}^{2}+z_{1}z_{2}^{*}},\;\beta_{21}=z_{1}^{*}z_{2} \end{array}$$

Note that, although $R_{ij}$ and $\beta_{ij}$ are complex numbers, the predictability P in Equation (125) is a real number.

#### 3.2.5. Visibility

The visibility is determined by the off-diagonal elements of the density matrix. Its direct calculation is pretty involved, which can be done in the following way. According to Equation (64), we have

(130) $$\begin{array}{cc} V^{2} & =\sum_{n\neq m}|\rho_{nm}^{\left(1\right)}{|}^{2}=2\sum_{n<m}|\rho_{nm}^{\left(1\right)}{|}^{2}=2\sum_{n=0}^{\infty }\sum_{m=n+1}^{\infty }{\left|\rho_{nm}^{\left(1\right)}\right|}^{2}=2\sum_{n=0}^{\infty }\left(\sum_{m=0}^{\infty }|\rho_{nm}^{\left(1\right)}{|}^{2}-\sum_{m=0}^{n}{\left|\rho_{nm}^{\left(1\right)}\right|}^{2}\right), \end{array}$$

where, according to Equation (119),

(131) $$|\rho_{nm}^{\left(1\right)}{|}^{2}=\alpha^{4}\sum_{ijkl}R_{ij}R_{kl}^{*}\frac{{\left(z_{i}z_{k}^{*}\right)}^{n}}{n!}\frac{{\left(z_{l}z_{j}^{*}\right)}^{n}}{m!}=\alpha^{4}\sum_{ijkl}R_{ij}R_{lk}\frac{\beta_{ik}^{n}}{n!}\frac{\beta_{lj}^{m}}{m!}.$$

(Note that we used $R_{kl}^{*}=R_{lk}$.)

As a result, we obtain

(132) $$\begin{array}{cc} V^{2} & =2\sum_{n=0}^{\infty }\left(\sum_{m=0}^{\infty }|\rho_{nm}^{\left(1\right)}{|}^{2}-\sum_{m=0}^{n}{\left|\rho_{nm}^{\left(1\right)}\right|}^{2}\right)=2\alpha^{4}\sum_{ijkl}R_{ij}R_{lk}\left[\sum_{n=0}^{\infty }\sum_{m=0}^{\infty }\frac{\beta_{ik}^{n}}{n!}\frac{\beta_{lj}^{m}}{m!}-\sum_{n=0}^{\infty }\sum_{m=0}^{n}\frac{\beta_{ik}^{n}}{n!}\frac{\beta_{lj}^{m}}{m!}\right]\\ \; & =2\alpha^{4}\sum_{ijkl}R_{ij}R_{lk}\left[e^{\beta_{ik}+\beta_{lj}}-e^{\beta_{lj}}\sum_{n=0}^{\infty }\frac{\beta_{ik}^{n}\Gamma \left(n+1,\beta_{lj}\right)}{{\left(n!\right)}^{2}}\right]\\ \; & =2\alpha^{4}\sum_{ijkl}R_{ij}R_{lk}\left[e^{\beta_{ik}+\beta_{lj}}-e^{\beta_{lj}}m\left(\beta_{ik},\beta_{lj}\right)\right], \end{array}$$

where we have used

(133) $$\sum_{m=0}^{n}\frac{x^{m}}{m!}=\frac{e^{x}\Gamma \left(n+1,x\right)}{n!}$$

with the incomplete Gamma function defined as $\Gamma \left(n+1,x\right)=\int_{x}^{\infty }t^{n}e^{-t}dt$, and we have also introduced a two-variable function

(134) $$m\left(x,y\right)=\sum_{n=0}^{\infty }\frac{x^{n}\Gamma \left(n+1,y\right)}{{\left(n!\right)}^{2}}.$$

The function m(x,y) has integral expressions in terms of the modified Bessel function given in Appendix A.

We have thus obtained the visibility in the Fock-state representation for quanton 1

(135) $$V=\alpha^{2}\sqrt{2\sum_{ijkl}R_{ij}R_{lk}\left[e^{\beta_{ik}+\beta_{lj}}-e^{\beta_{lj}}m\left(\beta_{ik},\beta_{lj}\right)\right]}.$$

#### 3.2.6. Local Complementarity Relation

To verify the local complementarity relation $P^{2}+V^{2}=Q^{2}$, we invoke the following property of the function m(x,y):

(136) $$e^{y}m\left(x,y\right)+e^{x}m\left(y,x\right)=e^{x+y}+I_{0}\left(2\sqrt{xy}\right),$$

which is derived in Appendix A. Then, according to the expression of V in Equation (135), we have

(137) $$\begin{array}{cc} V^{2} & =2\alpha^{4}\sum_{ijkl}R_{ij}R_{lk}\left[e^{\beta_{ik}+\beta_{lj}}-e^{\beta_{lj}}m\left(\beta_{ik},\beta_{lj}\right)\right]\\ \; & =\alpha^{4}\sum_{ijkl}R_{ij}R_{lk}\left[2e^{\beta_{ik}+\beta_{lj}}-e^{\beta_{lj}}m\left(\beta_{ik},\beta_{lj}\right)-e^{\beta_{ik}}m\left(\beta_{lj},\beta_{ik}\right)\right]\\ \; & =\alpha^{4}\sum_{ijkl}R_{ij}R_{lk}\left[2e^{\beta_{ik}+\beta_{lj}}-e^{\beta_{ik}+\beta_{lj}}-I_{0}\left(2\sqrt{\beta_{ik}\beta_{lj}}\right)\right]\\ \; & =\alpha^{4}\sum_{ijkl}R_{ij}R_{lk}\left[e^{\beta_{ik}+\beta_{lj}}-I_{0}\left(2\sqrt{\beta_{ik}\beta_{lj}}\right)\right]\\ \; & =\alpha^{4}\sum_{ijkl}R_{ij}R_{lk}e^{\beta_{ik}+\beta_{lj}}-\alpha^{4}\sum_{ijkl}R_{ij}R_{lk}I_{0}\left(2\sqrt{\beta_{ik}\beta_{lj}}\right)\\ \; & =Q^{2}-P^{2}, \end{array}$$

which is equivalent to the local complementarity relation $P^{2}+V^{2}=Q^{2}$. In the second step of the above derivation, we have made a switching of the index i↔l and j↔k. With the expressions of $R_{ij}$ and $\beta_{ij}$ in Equations (126)–(129), it is also easy to show $Q^{2}=\alpha^{4}\sum_{ijkl}R_{ij}R_{lk}e^{\beta_{ik}+\beta_{lj}}$ in the last step of the above derivation. Thus, we have verified the local complementarity relation in this example. (The difficulty in the direct evaluation of the visibility also highlights the usefulness of the local complementarity relation in the calculation of the visibility. Since Q and P are usually easier to calculate, the visibility can then be obtained indirectly by $V=\sqrt{Q^{2}-P^{2}}$.)

**Figure 6:**
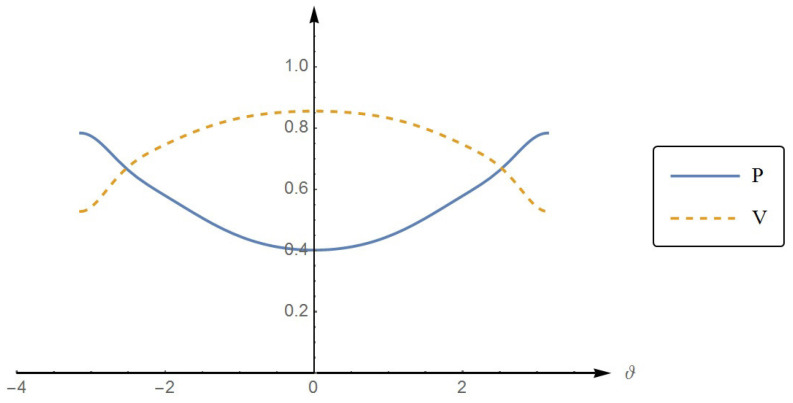
The graphs of the predictability P and the visibility V as functions of θ∈[−π,π] for the case with $z_{1}=1$ and $z_{2}=1+e^{i\theta }$. The purity Q in this case is constant, with the value Q≈0.945.

The local complementarity relation $P^{2}+V^{2}=Q^{2}$ shows that the predictability P (particle nature) and the visibility V (wave nature) are two complementary aspects of the purity Q (quantum nature). To visualize this complementarity relation in this particular example, we consider the more specific case with $z_{1}=1$ and $z_{2}=1+e^{i\theta }$ (θ∈[−π,π]). Then, in this case, $|z_{1}-z_{2}|=1$. According to the expression of Q in Equation (113), this implies

(138) $$Q=\frac{\sqrt{e^{-2}+6e^{-1}+1}}{\sqrt{2}\left(1+e^{-1}\right)}\approx 0.945,$$

independent of the variable θ. In other words, the purity Q is fixed when θ is varied. On the other hand, the predictability P and the visibility V are dependent on the variable θ. They are plotted as functions of θ in Figure 6. The complementarity between P and V as Q is fixed is manifested in their negative correlation seen from the figure.

In addition, the local complementarity relation $P^{2}+V^{2}=Q^{2}$ and the local-nonlocal complementarity relation $Q^{2}$ + $C^{2}/2$ = 1 together lead to the WPE complementarity relation $P^{2}$ + $V^{2}$ + $C^{2}/2$ = 1 for this example. We have thus verified the complementarity relations and obtained the expressions of all the relevant quantities for this particular example of an infinite-dimensional bipartite system in entangled coherent states.

## 4. Discussion

In this section, we discuss the connections and distinctions between our work and some relevant previous work [9,33]. The issue of measure matching is given particular attention.

### 4.1. Generalized Visibility and Predictability

Roy and Qureshi introduced in Reference [9] an alternative set of definitions for the generalized predictability and the generalized visibility (referred to as coherence and denoted by *C* there) for multi-slit interference experiments. They defined the predictability as

(139) $$P_{R}=\sqrt{1-{\left(\frac{1}{n-1}\sum_{i\neq j}\sqrt{\rho_{ii}\rho_{jj}}\right)}^{2}}$$

and the visibility as

(140) $$V_{R}=\frac{1}{n-1}\sum_{i\neq j}\left|\rho_{ij}\right|.$$

They have shown that

(141) $$P_{R}^{2}+V_{R}^{2}\leq 1,$$

where the equality holds for all pure states, obviously a desirable property. However, we would like to discuss one important difference with the results in this work in the following.

In comparison with the local complementarity relation $P^{2}+V^{2}=Q^{2}$ in the present work (which implies that $P^{2}+V^{2}\leq 1$ with the equality saturated by pure states), one may introduce the counterpart of Q in terms of $P_{R}$ and $V_{R}$, defined as

(142) $$Q_{R}=\sqrt{P_{R}^{2}+V_{R}^{2}}=\sqrt{1-{\left(\frac{1}{n-1}\sum_{i\neq j}\sqrt{\rho_{ii}\rho_{jj}}\right)}^{2}+{\left(\frac{1}{n-1}\sum_{i\neq j}\left|\rho_{ij}\right|\right)}^{2}}.$$

$Q_{R}$ is in the range [0,1]. $Q_{R}=1$ if ρ is a pure state and $Q_{R}=0$ if ρ is maximally mixed (i.e., ρ=I/n) as can be readily verified. (These properties of $Q_{R}$ imply $P_{R}^{2}+V_{R}^{2}\leq 1$ with the equal sign saturated by all pure states.) Therefore, it would seem that $Q_{R}$ may serve as an alternative measure of the degree of purity of the state ρ. In particular, in the special case n=2 for single qubits, $Q_{R}$ coincides with Q (represented geometrically by the length of the Bloch vector), which is a measure of purity that is invariant under local unitary transformations (i.e., independent of representations). However, for multi-dimensional systems with n>2, $Q_{R}$ in Equation (142) is in general *representation-dependent*. This is in contrast with the purity Q in this work that is representation-independent, as it is defined in terms of the usual purity $\mathrm{tr}(\rho^{2})$ (see Equation (31)).

To show that $Q_{R}$ is not representation-independent in general, it is sufficient to consider a particular example. Take for instance the following two three-dimensional density matrices

(143) $$\rho =\left[\begin{array}{ccc} \frac{1}{2} & 0 & 0\\ 0 & \frac{1}{2} & 0\\ 0 & 0 & 0 \end{array}\right],\;\rho^{\prime }=\left[\begin{array}{ccc} \frac{1}{2} & 0 & 0\\ 0 & \frac{1}{4} & \frac{1}{4}\\ 0 & \frac{1}{4} & \frac{1}{4} \end{array}\right].$$

ρ and $\rho^{\prime }$ are related by the unitary transformation $\rho^{\prime }=U\rho U^{†}$ with

(144) $$U=\left[\begin{array}{ccc} 1 & 0 & 0\\ 0 & \frac{1}{\sqrt{2}} & \frac{1}{\sqrt{2}}\\ 0 & \frac{1}{\sqrt{2}} & -\frac{1}{\sqrt{2}} \end{array}\right].$$

Therefore, ρ and $\rho^{\prime }$ may be considered as two different representations of the same density operator.

Direct calculation using Equation (142) yields

(145) $$Q_{R}\left(\rho \right)=\sqrt{1-{\left(\frac{1}{2}\right)}^{2}+0^{2}}=\frac{\sqrt{3}}{2}$$

and

(146) $$Q_{R}\left(\rho^{\prime }\right)=\sqrt{1-{\left(\sqrt{\frac{1}{8}}+\sqrt{\frac{1}{8}}+\sqrt{\frac{1}{16}}\right)}^{2}+{\left(\frac{1}{4}\right)}^{2}}=\frac{2-\sqrt{2}}{4},$$

demonstrating that $Q_{R}$ in general is not representation-independent as $Q_{R}\left(\rho \right)\neq Q_{R}\left(\rho^{\prime }\right)$ with $\rho^{\prime }$ = $U\rho U^{†}$.

Thus, the predictability $P_{R}$ and visibility $V_{R}$ in the multi-beam setting lose one important property compared to the two-beam setting. That is, they cannot be considered as two complementary aspects of one representation-independent property (invariant under local unitary transformations). In addition, since the entanglement for bipartite systems (e.g., quantified by the generalized concurrence C) is invariant under local unitary transformations, there is no obvious way to connect the predictability $P_{R}$ and visibility $V_{R}$ (they form a representation-dependent quantity $Q_{R}$) to the generalized concurrence C without introducing additional elements. As a result, there does not seem to be a direct way to generalize the WPE complementarity relation for pure states to multi-dimensional bipartite systems using the definitions of $P_{R}$ and $V_{R}$.

This problem can also be observed from the perspective of measure matching. The structure of the generalized concurrence for pure states, $C\propto \sqrt{1-\mathrm{tr}(\rho_{1}^{2})}$ (root trace square of the density operator), is naturally related to the $l_{2}$ norm of coherence defined as the visibility V in Equation (30) (root sum square of the off-diagonal elements of the density matrix). In contrast, the visibility $V_{R}$ in Equation (140) is the $l_{1}$ norm of coherence (sum of the absolute values of the off-diagonal elements of the density matrix). Therefore, the connection of $V_{R}$ (based on the $l_{1}$ norm) to C (naturally related to the $l_{2}$ norm) is complicated by the mismatch of different measures. This is another reason why there does not seem to be a direct generalization of the WPE complementarity relation in terms of $V_{R}$ and $P_{R}$. However, it does not mean that the visibility V (the $l_{2}$ norm of coherence) is superior to $V_{R}$ (the $l_{1}$ norm of coherence) in every way. In fact, if considered merely as a coherence measure (instead of focusing on its connection to the concurrence), V has a disadvantage compared to $V_{R}$ in that it is no longer a coherence monotone when subselection based on measurement outcomes is allowed for incoherent operations, as pointed out in Reference [35]. It is just that, as far as the relation between visibility and concurrence is concerned, the visibility V has a more natural connection to the concurrence C than $V_{R}$ does. This is the point of measure matching.

### 4.2. WPM Complementarity Relation

Zhang et al. [33] developed a WPM complementarity relation among wave, particle, and mixedness aspects for multi-dimensional systems. Their WPM complementarity relation is represented by the inequality

(147) $$P_{1g}^{2}+V_{R}^{2}+S_{L}\leq 1,$$

where $P_{1g}$ is the “one-guess bet” predictability capturing the particle aspect, defined as

(148) $$P_{1g}=\frac{n}{n-1}\max_{i}\left\{\rho_{ii}\right\}-\frac{1}{n-1},$$

$V_{R}$ is the $l_{1}$ measure of coherence (visibility) describing the wave aspect (the same as $V_{R}$ in Equation (140)), given by

(149) $$V_{R}=\frac{1}{n-1}\sum_{i\neq j}\left|\rho_{ij}\right|,$$

and $S_{L}$ is the linear entropy quantifying the mixedness aspect, with the expression

(150) $$S_{L}=\frac{n}{n-1}\left[1-\mathrm{tr}\left(\rho^{2}\right)\right].$$

In terms of the mixedness defined as $M=\sqrt{S_{L}}$ in the present work, the WPM complementarity relation in Equation (147) reads

(151) $$P_{1g}^{2}+V_{R}^{2}+M^{2}\leq 1.$$

It becomes an equality for two-dimensional systems (n=2), but in general remains an inequality for multi-dimensional systems (n>2).

In the present work, there is also a WPM complementarity relation, in the form of an equality:

(152) $$P^{2}+V^{2}+M^{2}=1.$$

This relation remains an equality for multi-dimensional systems (n≥2), in contrast with the WPM complementarity relation in Equation (151) in the form of an inequality except for n=2. The WPM complementarity relation in Equation (152) with $M^{2}$ replaced by $S_{L}$ was also mentioned in [33] in a remark, but considered to be a bit trivial.

From a mathematical point of view, the tight equality in Equation (152) places a much more stringent constraint among the wave, particle, and mixedness properties than the inequality in Equation (151). In this regard, the equality form of the WPM complementarity relation makes a stronger statement and thus may be more desirable. In fact, the inequality form of the WPM complementarity in Equation (151) can be *derived from* the equality form in Equation (152), by taking into account the distinctions of predictability and visibility measures ($P_{1g}$ versus P and $V_{R}$ versus V). The two lemmas used in [33] to derive the inequality form of the WPM complementarity are essentially statements of measure comparison: $P_{1g}\leq P$ (equivalent to lemma 1) and $V_{R}\leq V$ (equivalent to lemma 2). As a consequence, $P_{1g}^{2}+V_{R}^{2}+M^{2}\leq P^{2}+V^{2}+M^{2}=1$, producing the inequality in Equation (151). It is the adoption of smaller measures of predictability and visibility that reduced the equality in Equation (152) to an inequality in Equation (151). This also resonates with the point of measure matching discussed previously. Even though there may be multiple measures for an individual property which all seem to be appropriate, some measures may be more suitable (useful or powerful) than others when the property is considered in relation to others. In other words, the issue of measure matching arises when the *relations* between different qualities are of concern. Mismatched measures could reduce the power of the formulated relation that may otherwise offer more information. Our choice of the tangle instead of the squared concurrence as the entanglement measure in the formulation of the entanglement–mixedness relation also has to do with this issue.

Moreover, we would like to stress that the WPM complementarity relation, in its nature, is a single-partite (local) relation, as the predictability, the visibility, and the mixedness can all be defined in terms of the single-partite density operator without referring to another system. In contrast, the WPE complementarity relation for pure states (Equation (28))

(153) $$P_{k}^{2}+V_{k}^{2}+C^{2}/\nu_{k}^{2}=1$$

connects the local single-partite properties (predictability $P_{k}$ and visibility $V_{k}$) with the nonlocal bipartite property (concurrence C). It has a different physical content from the WPM complementarity relation, even though they have similar forms, especially when the equality form in Equation (152) is used. In addition, the WPM complementarity in Equation (152) holds regardless of whether the bipartite system is in a pure or mixed state. In contrast, the WPE complementarity in Equation (153), in general, no longer holds for mixed states, and is replaced by the more general WPEI complementarity relation $\tilde{P_{k}^{2}}+\tilde{V_{k}^{2}}+\left(\tau +B^{2}\right)/\bar{\nu_{k}^{2}}=1$.

## 5. Conclusions

In this paper, we have formulated the WPEI complementarity for general multi-dimensional bipartite systems in pure or mixed states, and we have further extended its range of applications to incorporate hierarchical bipartite systems and infinite-dimensional bipartite systems.

- For multi-dimensional bipartite systems in pure states, we developed the extended form of the WPE complementarity relation, $P_{k}^{2}+V_{k}^{2}+C^{2}/\nu_{k}^{2}=1$, from the local-nonlocal complementarity relation, $Q_{k}^{2}+C^{2}/\nu_{k}^{2}=1$, and the local complementarity relation, $P_{k}^{2}+V_{k}^{2}=Q_{k}^{2}$, using generalized measures of the relevant properties as summarized in Equations (29)–(33). The additional factor $1/\nu_{k}^{2}$ in the extended form reflects the asymmetry in the two subsystems when they have different dimensions.
- For multi-dimensional bipartite systems in mixed states, we formulated the generalized form of the WPEI complementarity relation, $\tilde{P_{k}^{2}}+\tilde{V_{k}^{2}}+\left(\tau +B^{2}\right)/\bar{\nu_{k}^{2}}=1$, together with the local-nonlocal complementarity relation, $\tilde{Q_{k}^{2}}+\left(\tau +B^{2}\right)/\bar{\nu_{k}^{2}}=1$, and the entanglement–mixedness relation, $\left(\tau +B^{2}\right)/\bar{\nu_{k}^{2}}=\tilde{M_{k}^{2}}$, with the help of the single-partite complementarity relations: $P^{2}+V^{2}=Q^{2}$, $Q^{2}+M^{2}=1$, and $P^{2}+V^{2}+M^{2}=1$. Compared to the case of two-qubit systems, the extended WPEI complementarity has the unequal-weight averages in place of the equal-weight averages, reflecting the possible difference in the subsystem dimensions. Moreover, the tangle has replaced the squared concurrence as the entanglement measure. These two measures agree for two-qubit systems, but differ for mixed states of general bipartite systems. We motivated and argued with the results in rank-2 states that the tangle gives a more powerful formulation of the WPEI complementarity. We also presented a graphical representation of all the relations relevant to the WPEI complementarity, shown in Figure 3.
- We further demonstrated how the WPEI complementarity can be applied to hierarchical bipartite systems (bipartite systems consisting of bipartite systems) and infinite-dimensional bipartite systems.
- The general formulation of the WPEI complementarity was illustrated with two specific examples worked out in detail. The first example is a qubit–qutrit system in rank-2 mixed states. The second example is a pair of infinite-dimensional quantons in entangled coherent states.
- We also discussed the relation of the present study to some previous work, with a focus on the measure matching issue in the formulation of the complementarity relations.

The WPEI complementarity formulated for general bipartite systems in pure or mixed states in this work has both theoretical significance and experimental implications. On the theoretical side, this general formulation of the WPEI complementarity demonstrates that the role of entanglement as well as ignorance in relation to the wave–particle duality is a general feature that rests on the basic structure of quantum mechanics and extends to (at least) general bipartite systems, including hierarchical and infinite-dimensional bipartite systems. It also hints at the intriguing possibility of rebuilding the foundations of quantum mechanics from a set of basic components that incorporates the WPEI complementarity principle. On the experimental side, the complementarity relations for finite-dimensional bipartite systems may be tested in multi-beam interference experiments, as the quanton-detector systems in these experiments can be analyzed in the context of general multi-dimensional bipartite systems. The particular example of entangled coherent states studied in this work may facilitate future experimental investigations on complementarity relations in infinite-dimensional bipartite systems.

## Abbreviations

The following abbreviations are used in this manuscript:

WPE	wave–particle–entanglement
WPEI	wave–particle–entanglement–ignorance
WPM	wave–particle–mixedness

## Appendix A. Properties of the Function *m*(*x*,*y*)

The function m(x,y) is defined as

(A1) $$m\left(x,y\right)=\sum_{n=0}^{\infty }\frac{x^{n}\Gamma \left(n+1,y\right)}{{\left(n!\right)}^{2}}.$$

Starting from the above definition, we found two integral expressions for the function m(x,y) in terms of the modified Bessel function:

(A2) $$m\left(x,y\right)=\int_{0}^{x}e^{x-y-z}I_{0}\left(2\sqrt{zy}\right)dz+e^{-y}I_{0}\left(2\sqrt{xy}\right)=e^{x}-\int_{0}^{y}e^{-z}I_{0}\left(2\sqrt{zx}\right)dz.$$

These two integral expressions imply the property

(A3) $$e^{y}m\left(x,y\right)+e^{x}m\left(y,x\right)=e^{x+y}+I_{0}\left(2\sqrt{xy}\right),$$

which is shown as follows.

From the first integral expression, we have

(A4) $$e^{y}m\left(x,y\right)=e^{y}\left[\int_{0}^{x}e^{x-y-z}I_{0}\left(2\sqrt{zy}\right)dz+e^{-y}I_{0}\left(2\sqrt{xy}\right)\right]=\int_{0}^{x}e^{x-z}I_{0}\left(2\sqrt{zy}\right)dz+I_{0}\left(2\sqrt{xy}\right).$$

From the second integral expression, we have

(A5) $$e^{x}m\left(y,x\right)=e^{x}\left[e^{y}-\int_{0}^{x}e^{-z}I_{0}\left(2\sqrt{zy}\right)dz\right]=e^{x+y}-\int_{0}^{x}e^{x-z}I_{0}\left(2\sqrt{zy}\right)dz.$$

Combining the two, we obtain Equation (A3).

## References

[1] Bohr N. (1928). The quantum postulate and the recent development of atomic theory. Nature.

[2] Wootters W.K., Zurek W.H. (1979). Complementarity in the double-slit experiment: Quantum nonseparability and a quantitive statement of Bohr’s principle. Phys. Rev. D.

[3] Greenberger D.M., Yasin A. (1988). Simultaneous wave and particle knowledge in a neutron interferometer. Phys. Lett. A.

[4] Jaeger G., Shimony A., Vaidman L. (1995). Two interferometric complementarities. Phys. Rev. A.

[5] Englert B.G. (1996). Fringe visibility and which-way information: An inequality. Phys. Rev. Lett..

[6] Englert B.G., Bergou J.A. (2000). Quantitative quantum erasure. Opt. Commun..

[7] Dürr S. (2001). Quantitative wave–particle duality in multibeam interferometers. Phys. Rev. A.

[8] Englert B.G., Kaszlikowski D., Kwek L.C., Chee W.H. (2008). Wave–particle duality in multi-path interferometers: General concepts and three-path interferometers. Int. J. Quantum Inf..

[9] Roy P., Qureshi T. (2019). Path predictability and quantum coherence in multi-slit interference. Phys. Scr..

[10] Bera M.N., Qureshi T., Siddiqui M.A., Pati A.K. (2015). Duality of quantum coherence and path distinguishability. Phys. Rev. A.

[11] Siddiqui M.A., Qureshi T. (2015). Three-slit interference: A duality relation. Prog. Theor. Exp. Phys..

[12] Bagan E., Bergou J.A., Cottrell S.S., Hillery M. (2016). Relations between coherence and path information. Phys. Rev. Lett..

[13] Coles P.J. (2016). Entropic framework for wave–particle duality in multipath interferometers. Phys. Rev. A.

[14] Qureshi T., Siddiqui M.A. (2017). Wave–particle duality in N-path interference. Ann. Phys..

[15] Jakob M., Bergou J.A. (2010). Quantitative complementarity relations in bipartite systems: Entanglement as a physical reality. Opt. Commun..

[16] Fedrizzi A., Skerlak B., Paterek T., de Almeida M.P., White A.G. (2011). Experimental information complementarity of two-qubit states. New J. Phys..

[17] Kaiser F., Coudreau T., Milman P., Ostrowsky D.B., Tanzilli S. (2012). Entanglement-enabled delayed-choice experiment. Science.

[18] Banaszek K., Horodecki P., Karpinski M., Radzewicz C. (2013). Quantum mechanical which-way experiment with an internal degree of freedom. Nat. Commun..

[19] Prabhu Tej J., Usha Devi A.R., Karthik H.S., Sudha, Rajagopal A.K. (2014). Quantum which-way information and fringe visibility when the detector is entangled with an ancilla. Phys. Rev. A.

[20] Tessier T.E. (2005). Complementarity relations for multi-qubit systems. Found. Phys. Lett..

[21] Qian X.-F., Vamivakas A.N., Eberly J.H. (2018). Entanglement limits duality and vice versa. Optica.

[22] Eberly J.H., Qian X.-F., Vamivakas A.N. (2017). Polarization coherence theorem. Optica.

[23] Qian X.-F., Malhotra T., Vamivakas A.N., Eberly J.H. (2016). Coherence constraints and the last hidden optical coherence. Phys. Rev. Lett..

[24] Peng X., Zhu X., Suter D., Du J., Liu M., Gao K. (2005). Quantification of complementarity in multiqubit systems. Phys. Rev. A.

[25] Peng X., Zhang J., Du J., Suter D. (2008). Quantitative complementarity between local and nonlocal character of quantum states in a three-qubit system. Phys. Rev. A.

[26] Rungta P., Buzek V., Caves C.M., Hillery M., Milburn G.J. (2001). Universal state inversion and concurrence in arbitrary dimensions. Phys. Rev. A.

[27] Rungta P., Caves C.M. (2003). Concurrence-based entanglement measures for isotropic states. Phys. Rev. A.

[28] Mintert F., Kuś M., Buchleitner A. (2004). Concurrence of mixed bipartite quantum states in arbitrary dimensions. Phys. Rev. Lett..

[29] Englert B.-G., Aharonov Y. (2001). The mean king’s problem: Prime degrees of freedom. Phys. Lett. A.

[30] Coffman V., Kundu J., Wootters W.K. (2000). Distributed entanglement. Phys. Rev. A.

[31] Osborne T.J. (2005). Entanglement measure for rank-2 mixed states. Phys. Rev. A.

[32] Hill S., Wootters W.K. (1997). Entanglement of a pair of quantum bits. Phys. Rev. Lett..

[33] Zhang X., Huang J., Zhuang M., Qin X., Lee C. (2017). Wave–particle-mixedness complementarity. arXiv.

[34] Sanders B.C. (2012). Review of entangled coherent states. J. Phys. A Math. Theor..

[35] Baumgratz T., Cramer M., Plenio M.B. (2014). Quantifying coherence. Phys. Rev. Lett..

